# Supplementary material for: Evolutionary patterns of the SSU rRNA (V4 region) secondary structure in genus Euplotes (Ciliophora, Spirotrichea): insights into cryptic species and primitive traits
Source: PeerJ. 2025 Jan 23;13:e18852. doi: 10.7717/peerj.18852 (PMC11766670; doi:10.7717/peerj.18852)
Supplement: Supplemental Information 1 [file peerj-13-18852-s001.docx]

| **Clade** | **Species** | **Accession number**  **(SSU)** | **V4 region length**  **(nt)** | **Free energy**  **(Joule)** | **Secondary structure type** |
| --- | --- | --- | --- | --- | --- |
| I | *E. sinicus* | FJ423448 | 174 | -54.10 | I |
|  | *E. petzi* | KJ434104 | 178 | -53.10 | I |
|  | *E. huizhouensis* | MW164888 | 174 | -48.20 | I |
| II | *E. warreni* | MT742131 | 169 | -66.10 | I |
|  | *E. parabalteatus* | FJ346568 | 180 | -62.20 | I |
|  | *E. weissei* | MN593323 | 181 | -55.40 | I |
|  | *E. dammamensis* | JX185743 | 180 | -55.50 | I |
| III | *E. elegans* | DQ309868 | 248 | -84.98 | II |
|  | *E. wuhanensis* | MH795291 | 261 | -73.36 | II |
|  | *E. bergeri* | MN593322 | 253 | -65.78 | II |
|  | *E. qatarensis* | KU555390 | 255 | -79.82 | II |
|  | *E. dominicanus* | MN757874 | 173 | -64.80 | I |
|  | *E. estuarinus* | MF445655 | 174 | -61.60 | I |
|  | *E. curdsi* | KY855580 | 174 | -61.60 | I |
|  | *E. nobili* | JQ003922 | 174 | -66.50 | I |
|  | *E. shii* | MW164887 | 174 | -61.90 | I |
|  | *E. raikovi* | KX516720 | 174 | -65.20 | I |
| IV | *E. parawoodruffi* | AF452708 | 202 | -62.07 | II |
|  | *E. woodruffi* | AF452710 | 202 | -62.07 | II |
|  | *E. aediculatus* | FR873712 | 202 | -63.37 | II |
|  | *E. paramieti* | OM65848 | 205 | -62.23 | II |
|  | *E. amieti* | KJ524911 | 205 | -56.95 | II |
|  | *E. eurystomus* | FR873716 | 205 | -56.95 | II |
|  | *E. patella* | EF094964 | 211 | -53.25 | II |
|  | *E. octocarinatus* | EF094962 | 210 | -55.99 | II |
|  | *E. daidaleos* | FR873718 | 206 | -60.23 | II |
|  | *E. neapolitanus* | FJ998024 | 205 | -61.65 | II |
|  | *E. platystoma* | MF928801 | 223 | -57.56 | II |
|  | *E. harpa* | AJ305252 | 203 | -57.56 | II |
| V | *E. rariseta* | FJ423449 | 197 | -47.72 | II |
|  | *E. muscicola* | MH795290 | 192 | -52.44 | II |
|  | *Euplotes* sp. | PP648200 | 192 | -53.54 | II |
|  | *Euplotes* n. sp | PP648199 | 192 | -52.44 | II |
|  | *E. paramuscicola* | PP648196 | 200 | -52.15 | II |
|  | *E. lynni* | MG827339 | 195 | -46.43 | II |
|  | *E. muscorum* | DQ661046 | 196 | -48.82 | II |
|  | *E. novemcarinatus* | HM140402 | 195 | -54.33 | II |
|  | *E. gracilis* | PP648194 | 195 | -54.33 | II |
|  | *E. muscorum oligomembrana* n. subsp. | PP648195 | 196 | -52.62 | II |
|  | *E. encysticus* | EF535728 | 195 | -54.33 | II |
|  | *E. foissneri* | MT742130 | 190 | -60.97 | II |
|  | *E. chongmingensis* | OM065849 | 199 | -62.72 | II |
|  | *E.* cf. *trisulcatus* | PP648193 | 199 | -60.92 | II |
|  | *E. indica* | MN038061 | 199 | -62.72 | II |
|  | *E. euryhalinus* | MG994991 | 199 | -62.72 | II |
|  | *E. magnicirratus* | AJ549210 | 191 | -58.15 | II |
|  | *E.* cf*. inkystans* | PP648189 | 191 | -58.15 | II |
|  | *E. trisulcatus* | EF690810 | 196 | -58.14 | I |
|  | *E.* cf*. antarcticus* | FJ998023 | 198 | -46.07 | II |
|  | *E. balteatus* | JX185744 | 190 | -67.03 | II |
|  | *E. plicatum* | EF094966 | 190 | -76.53 | II |
|  | *E. orientalis* | KX516666 | 190 | -64.53 | II |
|  | *E. enigma* | LT732572 | 190 | -62.13 | II |
|  | *E. alatus* | KJ434102 | 190 | -62.13 | II |
|  | *E. bisulcatus* | EF094965 | 189 | -54.20 | I |
|  | *E. shini* | MN593321 | 192 | -54.20 | I |
|  | *E. focardii* | EF094960 | 207 | -42.97 | II |
|  | *E. quinquecarinatus* | JX437136 | 208 | -44.85 | II |
|  | *E. parkei* | AJ305247 | 201 | 46.89 | II |
|  | *E. charon* | FJ870078 | 200 | -46.95 | II |
| VI | *E. minuta* | AJ305244 | 196 | -51.47 | II |
|  | *E. cristatus* | GU953667 | 187 | -52.80 | I |
|  | *E. vannus* | AY361854 | 194 | -52.58 | I |
|  |  | AY004772 | 191 | -54.60 | I |
|  | *E. crassus* | KX516710 | 193 | -52.61 | I |
|  |  | AY361863 | 189 | -58.40 | I |
|  | *E. japonicum* | ON387646 | 190 | -54.60 | I |
|  | *E. crenosus* | PP648191 | 190 | -54.60 | I |
|  | *E*. cf. *mutabilis* | PP648190 | 192 | -54.60 | I |
| Outgroup | *Aspadisca fusca*  *Certesia quadrinucleata*  *Discocephalus pararotatorius*  *Diophrys scutum*  *Euplotidium Rosati*  *Uronychia xinjiangensis* | JX025168  DQ059581  JX460983  EU189069  HE775112  KX147287 | 165  165  166  164  163  163 | -52.20  -61.70  -44.30  -47.70  -33.30  -39.60 | I  I  I  I  I  I |
