# Supplementary material for: Evolutionary patterns of the SSU rRNA (V4 region) secondary structure in genus Euplotes (Ciliophora, Spirotrichea): insights into cryptic species and primitive traits
Source: PeerJ. 2025 Jan 23;13:e18852. doi: 10.7717/peerj.18852 (PMC11766670; doi:10.7717/peerj.18852)
Supplement: Supplemental Information 2 [file peerj-13-18852-s002.docx]

| **Clade** | **Species** | **Accession number**  **(SSU)** | **Origin** | **Publication** |
| --- | --- | --- | --- | --- |
| I | *E. sinicus* | FJ423448 | Qingdao, China | Jiang et al., 2010a |
|  | *E. petzi* | KJ434104 | Arctic | Di Giuseppe et al., 2014 |
|  |  | KJ434106 | Antarctic |  |
|  | *E. huizhouensis* | MW164888 | Huizhou, China | Lian et al., 2021 |
| II | *E. warreni* | MT742131 | Punta Arenas, Chile | Valbonesi et al., 2021 |
|  | *E. parabalteatus* | KX516723 | Zhanjiang, China | Zhao et al., 2018 |
|  |  | FJ346568 | Qingdao, China | Yi et al., 2009 |
|  | *E. weissei* | MN593323 | Qingdao, China | Lian et al., 2020 |
|  | *E. dammamensis* | JX185743 | Arabian Gulf, Saudi Arabia | Chen et al., 2013 |
| III  III | *E. elegans* | DQ309868 | Mariager Fjord, Denmark | Julian Schwarz et al., 2007 |
|  | *E. wuhenensis* | MH795291 | Wuhan, China | Lian et al., 2019 |
|  | *E. dominicanus* | MN757874 | North Atlantic | Živaljić et al., 2020 |
|  | *E. estuarinus* | MF445655 | Guangzhou, China | Yan et al., 2018 |
|  | *E. curdsi* | KX819312 | Agrigento, Italy | Syberg-Olsen et al., 2016 |
|  |  | KX819313 | Sredny Island, Russia |  |
|  |  | KY855580 | Andhra Pradesh, India | - |
|  | *E. nobilii* | JQ003922 | Siorapaluk, Greenland | Di Giuseppe et al., 2013 |
|  |  | KP843612 | Barrow, Alaska USA | Di Giuseppe et al., 2015 |
|  | *E. shii* | MW164887 | Huizhou, China | Lian et al., 2021 |
|  | *E. raikovi* | FJ423447 | Qingdao, China | Jiang et al., 2010b |
|  |  | KX516720 | Zhanjiang, China | Zhao et al., 2018 |
|  | *E. bergeri* | MN593322 | Huizhou, China | Lian et al., 2020 |
|  | *E. qatarensis* | KU555390 | inland Sea Khor al Adaid, Qatar | Fotedar et al., 2019 |
| IV  IV  IV | *E. parawoodruffi* | FJ998025 | Qingdao, China | - |
|  |  | AF452708 |  |  |
|  | *E. woodruffi* | KX516657 | Shenzhen, China | Zhao et al., 2018 |
|  |  | KY855578 | Andhra Pradesh, India | - |
|  |  | LT623906 | Italy | - |
|  |  | AF452710 | Qingdao, China | - |
|  |  | JQ801445 | Putian, China | Dai et al., 2013 |
|  |  | JQ801446 |  |  |
|  |  | EF193244 | Comacchio, Italy | Fokin et al., 2008 |
|  | *E. aediculatus* | FR873712 | Pisa, Italy | Vannini et al., 2012 |
|  |  | FR873713 | Delhi (India) |  |
|  |  | AJ305253 | Stirone river, Italy | Petroni et al., 2002 |
|  | *E. paramieti* | OM065848 | Shanghai, China | Han et al., 2022 |
|  | *E. amieti* | KJ524911 | Shanghai, China | Liu et al., 2015 |
|  | *E. eurystomus* | EF193250 | Pisa, Italy | Fokin et al., 2008 |
|  |  | FR873716 |  | Vannini et al., 2012 |
|  | *E. patella* | EF094964 | Hakusui, Japan | Vallesi et al., 2008 |
|  | *E.octocarinatus* | MK411264 | Oaxaca, mexico | Méndez-Sánchez et al., 2020 |
|  |  | EF094962 | Pisa, Italy | Vallesi A. *et al*., 2008 |
|  |  | AJ310489 | Mu¨ nster, Germany | Bernhard et al., 2001 |
|  | *E. daidaleos* | EF690811 | Kaiserslautern, Germany | - |
|  |  | FR873718 | Pisa, Italy | Vannini et al., 2012 |
|  |  | KF887346 | Dorset, UK | - |
|  |  | AM930379 | Argyll, UK | Achilles-Day et al., 2008 |
|  | *E. platystoma* | MF928801 | Shenzhen, China | Lian et al., 2018 |
|  |  | MF445654 | Huizhou, China | Yan et al., 2018 |
|  | *E. neapolitanus* | FJ998024 | Huizhou, China | Liu et al., 2020 |
|  |  | KX516656 | Shenzhen, China | Zhao et al., 2018 |
|  | *E. harpa* | KX516655 | Shenzhen, China | Zhao et al., 2018 |
|  |  | HM140408 | Ulsan, Korea | - |
|  |  | AJ305252 | Bornholm, Baltic Sea | Petroni et al., 2002 |
|  |  | AJ811015 | Pisa, Italy | Vannini et al., 2005 |
|  |  | AJ811016 | Puglia, Italy |  |
|  | *E. rariseta* | MK050525 | Marine sediment, Korea | - |
|  |  | FJ423449 | Qingdao, China | Jiang et al., 2010a |
|  |  | AJ305248 | Garachico, Spain | Petroni et al., 2002 |
| V  V  V | *E. muscicola* | AJ305254 | Bagnone, Italy | Petroni et al., 2002 |
|  |  | MH795290 | Wuhan, China | Lian et al., 2019 |
|  | *E. lynni* | MG827339 | Delhi, India | Abraham et al., 2021 |
|  | *E. muscorum* | DQ661046 | Baltimore, USA | - |
|  |  | HM140407 | Ulsan, Korea | - |
|  | *E. novemcarinatus* | HM140402 | Ulsan, Korea | - |
|  | *E. encysticus* | KY855579 | Andhra Pradesh, India | - |
|  |  | KX516673 | Shenzhen, China | Zhao et al., 2018 |
|  |  | MK026950 | Dokdo, Korea | - |
|  |  | LN864512 | Saquarema, Brazil | Senra et al., 2016 |
|  |  | LT628496 | Andhra Pradesh, India | - |
|  |  | KY922820 | Wuhan, China | - |
|  |  | EF535728 | Shanghai, China | - |
|  |  | FJ346569 | Qingdao, China | Yi et al., 2009 |
|  | *E. foissneri* | MT742130 | Puerto Natales, Chile | Valbonesi et al., 2021 |
|  | *E. chongmingensis* | OM065849 | Shanghai, China | Han et al., 2022 |
|  | *E. indica* | MN038061 | Delhi, India | Abraham et al., 2021 |
|  | *E. euryhalinus* | MG994991 | Delhi, India | - |
|  |  | JF903799 | Andhra Pradesh, India | - |
|  |  | KP297363 | Pisa, Italy | - |
|  |  | KP297367 |  |  |
|  |  | KP297368 |  |  |
|  | *E. magnicirratus* | KX516685 | Shantou, China | Zhao et al., 2018 |
|  |  | AJ549210 | Campionna, Italy | Petroni et al., 2002 |
|  |  | AJ305250 | Caribbean Sea, Columbia | Petroni et al., 200 |
|  | *E.* cf*. antarcticus* | FJ998023 | Huizhou, China | Liu et al., 2020 |
|  |  | KX516668 | Shenzhen, China | Zhao et al., 2018 |
|  | *E. trisulcatus* | EF690810 | Kaiserslautern, Germany | - |
|  | *E. balteatus* | JX185744 | Arabian Gulf, Saudi Arabia | Chen et al., 2013 |
|  | *E. orientalis* | KX516666 | Zhanjiang, China | Zhao et al., 2018 |
|  |  | FJ875138 | Qingdao, China | Jiang et al., 2010b |
|  | *E. plicatum* | EF094966 | Christchurch, New Zealand | Vallesi et al., 2008 |
|  | *E. enigma* | LT732572 | Victoria, BC, Canada | Boscaro et al., 2019 |
|  | *E. alatus* | KJ434102 | Pisa, Italy | Di Giuseppe et al., 2014 |
|  | *E. bisulcatus* | EF094965 | Christchurch, New Zealand | Vallesi et al., 2008 |
|  | *E. shini* | MN593321 | Huizhou, China | Lian et al., 2020 |
|  | *E. focardii* | EF094960 | Antarctica | Vallesi et al., 2008 |
|  | *E. quinquecarinatus* | JX437136 | Mughsayl, Oman | Guella et al., 2012 |
|  | *E. parkei* | AJ305247 | Venezuela | Petroni et al., 2002 |
|  | *E. charon* | KX516686 | Qingdao, China | Zhao et al., 2018 |
|  |  | JF694043 |  | Huang et al., 2012 |
|  |  | KX516688 |  | Zhao et al., 2018 |
|  |  | FJ870078 |  | - |
|  |  | KX516691 |  | Zhao et al., 2018 |
|  |  | FJ870080 |  | - |
|  |  | KX516698 | Hongkong, China | Zhao et al., 2018 |
| VI  VI | *E. minuta* | KX516699 | Qingdao, China | Zhao et al., 2018 |
|  |  | EF094958 | Shetland, UK | Vallesi et al., 2008 |
|  |  | AJ305244 | Maratea, Italy | Petroni et al., 2002 |
|  |  | GU953668 | Incheon, Korea | Park et al., 2010 |
|  | *E. cristatus* | GU953667 | Incheon, Korea | Park et al., 2010 |
|  | *E. vannus* | EF094956 | Hurghada, Egypt | Vallesi et al., 2008 |
|  |  | AY361854 | Pisa, Italy | - |
|  |  | MG603631 | Incheon, Korea | Park et al., 2019 |
|  |  | KY922821 | Wuhan, China | Yuan et al., 2018 |
|  |  | AY004772 | Qingdao, China | Chen et al., 2002 |
|  |  | KX516701 | Shenzhen, China | Zhao et al., 2018 |
|  | *E. crasssus* | KX516710 | Shenzhen, China | Zhao et al., 2018 |
|  |  | EF094954 | Portugal | Vallesi et al., 2008 |
|  |  | HQ413693 | Incheon, Korea | Kim et al., 2011 |
|  |  | AJ305239 | Setubal, Portugal | Petroni et al., 2002 |
|  |  | AY361863 | Pisa, Italy | - |
|  | *E. japonicum* | ON387646 | The peter Great Gulf, japan | Mikhail et al., 2022 |

**References:**

Bernhard D, Stechmann A, Foissner W, Ammermann D, Hehn M, Schlegel M. 2001. Phylogenetic relationships within the class Spirotrichea (Ciliophora) inferred from small subunit rRNA gene sequences. *Molecular Phylogenetics and Evolution* 21(1):86–92

DOI 10.1006/mpev.2001.0997.

Chen Z, Song W. 2002. Phylogenetic positions of *Aspidisca steini* and *Euplotes vannus* within the order Euplotida (Hypotrichia, Ciliophora) inferred from complete small subunit ribosomal RNA gene sequences. *Acta Protozoologica* 41(1):1–10.

Chen X, Zhao Y, Al-Farraj SA, Al-Quraishy S, El-Serehy HA, Shao C, Al-Rasheid KA. 2013. Taxonomic descriptions of two marine ciliates *Euplotes dammamensis* n. sp. And *Euplotes balteatus (*Dujardin, 1841) Kahl, 1932 (Ciliophora, Spirotrichea, Euplotida), collected from the Arabian Gulf, Saudi Arabia. *Acta Protozoologica* 52(2):73–89 DOI 10.4467/16890027AP.13.008.1087.

Dai R, Xu K, He Y. 2013. Morphological, physiological, and molecular evidences suggest that *Euplotes parawoodrufﬁ* is a junior synonym of *Euplotes woodrufﬁ* (Ciliophora Euplotida). *Journal of Eukaryotic Microbiology* 60(1):70–78 DOI 10.1111/jeu.1207.

Di Giuseppe G, Barbieri M, Vallesi A, Luporini P, Dini F. 2013. Phylogeographical pattern of *Euplotes nobilii,* a protist ciliate with a bipolar biogeographical distribution. *Molecular Ecology* 22(15):4029–4037 DOI 10.1111/mec.12363.

Di Giuseppe G, Dini F, Vallesi A, Luporini P. 2015. Genetic relationships in bipolar species of the protist ciliate *Euplotes*. *Hydrobiologia* 761(1):71–83 DOI 10.1007/s10750-015-2274-5.

Di Giuseppe G, Erra F, Frontini FP, Dini F, Vallesi A, Luporini P. 2014. Improved description of the bipolar ciliate *Euplotes petzi* and deﬁnition of its basal position in the *Euplotes* phylogenetic tree. *European Journal of Protistology* 50(4):402–411

DOI 10.1016/j.ejop.2014.05.001.

Fokin SI, Di Giuseppe G, Erra F, Dini F. 2008. *Euplotespora binucleatan*. gen., n. sp. (Protozoa: Microsporidia), a parasite infecting the hypotrichous ciliate *Euplotes woodrufﬁ*, with observations on microsporidian infections in Ciliophora. *Journal of Eukaryotic Microbiology* 55(3):214–228 DOI 10.1111/j.1550-7408.2008.00322.x.

Fotedar R, Stoeck T, Filker S, Fell JW, Agatha S, Al Marri M, Jiang J. 2016. Description of the halophile *Euplotes qatarensis* nov. spec. (Ciliophora, Spirotrichea, Euplotida) isolated from the hypersaline Khor Al-Adaid Lagoon in Qatar. *Journal of Eukaryotic Microbiology* 63(5):578–590 DOI 10.1111/jeu.12305.

Guella G, Callone E, Mancini I, Dini F, Di Giuseppe G. 2012. Diterpenoids from marine ciliates: chemical polymorphism of *Euplotes rariseta*. *European Journal of Organic Chemistry* 2012 (27):5208–5216 DOI 10.1002/ejoc.201200559.

Huang J, Dunthorn M, Song W. 2012. Expanding character sampling for the molecular phylogeny of euplotid ciliates (Protozoa, Ciliophora) using three markers, with a focus on the family Uronychiidae. *Molecular Phylogenetics and Evolution* 63(3):598–605 DOI 10.1016/j.ympev.2012.02.007.

Jiang J, Zhang Q, Hu X, Shao C, Al-Rasheid KA, Song W. 2010a.Two new marine ciliates, *Euplotes sinicus* sp. nov. and *Euplotes parabalteatus* sp. nov., and a new small subunit rRNA gene sequence of *Euplotes rariseta* (Ciliophora, Spirotrichea, Euplotida). *International Journal of Systematic and Evolutionary* Microbiology 60(5):1241–1251 DOI 10.1099/ijs.0.012120-0.

Jiang J, Zhang Q, Warren A, Al-Rasheid KA, Song W. 2010b. Morphology and SSU rRNA gene-based phylogeny of two marine *Euplotes* species *E. orientalis* spec. nov. and *E. raikovi* (Ciliophora, Euplotida). *European Journal of Protistology* 46(2):121–132 DOI 10.1016/j.ejop.2009.11.003.

Julian Schwarz MV, Zuendorf A, Stoeck T. 2007.Morphology, ultrastructure, molecular phylogeny, and autecology of *Euplotes elegans* Kahl, 1932 (Hypotrichida; Euplotidae) isolated from the anoxic Mariager Fjord, Denmark. *Journal of Eukaryotic Microbiology* 54(2):125–136 DOI 10.1111/j.1550-7408.2007.00243.x.

Lian C, Luo X, Fan X, Huang J, Yu Y, Bourland W, Song W. 2018. Morphological and molecular redeﬁnition of *Euplotes platystoma* Dragesco & Dragesco-Kernéis, 1986 and *Aspidisca lynceus* (Müller, 1773) Ehrenberg, 1859, with reconsideration of a well-known *Euplotes* ciliate, *Euplotes harpa* Stein, 1859 (Ciliophora, Euplotida). *Journal of Eukaryotic Microbiology* 65(4):531–543

DOI 10.1111/jeu.12499.

Lian C, Wang Y, Jiang J, Yuan Q, Al-Farraj SA, El-Serehy HA, Song W, Stoeck T, Shao C. 2021. Systematic positions and taxonomy of two new ciliates found in China: *Euplotes tuffraui* sp. nov. and *E. shii* sp. nov. (Alveolata, Ciliophora, Euplotida). *Systematics and Biodiversity* 19 (4):359–374 DOI 10.1080/14772000.2020.1865472.

Liu W, Jiang J, Tan Y, Lin X. 2020. Novel contributions to the taxonomy of the Ciliates genus *Euplotes* (Ciliophora, Euplotida): redescription of two poorly known species, with a brief note on the distributions of this genus in coastal waters of southern China. *Frontiers in Marine Science* 7:615413 DOI 10.3389/fmars.2020.615413.

Méndez-Sánchez D, Mayén-Estrada R, Hu X. 2020. *Euplotes octocarinatus* Carter, 1972 (Ciliophora, Spirotrichea, Euplotidae): considerations on its morphology, phylogeny, and biogeography. *European Journal of Protistology* 74(2):125667

DOI 10.1016/j.ejop.2019.125667.

Mikhail T. 2022. Morphology and molecular phylogeny of *Euplotes japonicum* sp. n. (Ciliophora, Euplotidae) from the Peter the Great Gulf, Sea of Japan. *Protistology* 16(4):266–273 DOI 10.21685/1680-0826-2022-16-4-2.

Park MH, Kim SJ, Min GS. 2010. First record of two *Euplotes* Ciliates (Ciliophora: Spirotrichea: Euplotida) from Korea. *Animal Systematics, Evolution, and Diversity* 26(1):21–27 DOI 10.5635/KJSZ.2010.26.1.021.

Petroni G, Dini F, Verni F, Rosati G. 2002. A molecular approach to the tangled intrageneric relationships underlying phylogeny in Euplotes (Ciliophora, Spirotrichea). *Molecular Phylogenetics and Evolution* 22(1):118–130 DOI 10.1006/mpev.2001.1030.

Senra MV, Dias RJ, Castelli M, Silva-Neto ID, Verni F, Soares CA, Petroni G. 2016. A house for two double bacterial infection in *Euplotes woodrufﬁ* Sq1 (Ciliophora, Euplotia) sampled in Southeastern Brazil. *Microbial Ecology* 71(2):505–517 DOI 10.1007/s00248-015-0668-6.

Vallesi A, Di Giuseppe G, Dini F, Luporini P. 2008. Pheromone evolution in the protozoan ciliate, *Euplotes*: the ability to synthesize diffusible forms is ancestral and secondarily lost. *Molecular Phylogenetics and Evolution* 47(1):439–442

DOI 10.1016/j.ympev.2007.11.025.

Vannini C, Ferrantini F, Ristori A, Verni F, Petroni G. 2012. Betaproteobacterial symbionts of the Ciliate *Euplotes*: origin and tangled evolutionary path of an obligate microbial association. *Environmental Microbiology* 14(9):2553–2563

DOI 10.1111/j.1462-2920.2012.02760.x.

Vannini C, Petroni G, Verni F, Rosati G. 2005.Polynucleobacter bacteria in the brackish-water species *Euplotes harpa* (Ciliata Hypotrichia). *Journal of Eukaryotic Microbiology* 52(2):116–122

DOI 10.1111/j.1550-7408.2005.04-3319.x.

Yan Y, Fan Y, Luo X, El-Serehy HA, Bourland W, Chen X. 2018. New contribution to the species-rich genus *Euplotes*: morphology, ontogeny, and systematic position of two species (Ciliophora; Euplotia). *European Journal of Protistology* 64(5):20–39

DOI 10.1016/j.ejop.2018.03.003.

Yi Z, Song W, Clamp JC, Chen Z, Gao S, Zhang Q. 2009. Reconsideration of systematic relationships within the order Euplotida (Protista, Ciliophora) using new sequences of the gene coding for small-subunit rRNA and testing the use of combined data sets to construct phylogenies of the Diophrys-complex. *Molecular Phylogenetics and Evolution* 50(3):599–607

DOI 10.1016/j.ympev.2008.12.006.

Yuan D, Zhan X, Wang M, Wang X, Feng W, Gong Y, Hu Q. 2018. Biodiversity and distribution of microzooplankton in Spirulina (Arthrospira) platensis mass cultures throughout China. *Algal Research* 30:38–49 DOI 10.1016/j.algal.2017.12.009.

ŽivaljićS, Scherwass A, Schoenle A, Hohlfeld M, Quintela-Alonso P, Nitsche F, Arndt H. 2020. A barotolerant ciliate isolated from the abyssal deep sea of the North Atlantic: *Euplotes dominicanus* sp. n. (Ciliophora, Euplotia). *European Journal of Protistology* 73:125664

DOI 10.1016/j.ejop.2019.125664.
