# Supplementary material for: Evolutionary patterns of the SSU rRNA (V4 region) secondary structure in genus Euplotes (Ciliophora, Spirotrichea): insights into cryptic species and primitive traits
Source: PeerJ. 2025 Jan 23;13:e18852. doi: 10.7717/peerj.18852 (PMC11766670; doi:10.7717/peerj.18852)
Supplement: Supplemental Information 3 [file peerj-13-18852-s003.docx]

>1 Euplotes cf inkystans_ PP648189

TTGGCCTGGGCTGGGGAAGCATGCATGTACCACTATAAAGGAACATACAACAGAAACTGCGAATGGCTCATTCAAACAGTTATAGTTTATTTGATATTCAAACTTATATTCTCATATTAGTTTAAATGGATAACCGTAGTAATTCTAGAGCTAATACATGCGTTACGGGGGACTTTTCGGAACCTCAGTATTTATTAGATTATTAAACCAGCATTCCGCAAGGTCTACTGAGATGATTCATGATAACTGATCGAATTGCCGCGACTGTCGGCAATAAATCATTCATGTTTCTGCTTCCCATCAGCTCGATGGTAGTGTATTGGACAACCATGGCATTCACGGGCTATCGGGGGATTAGGGTTCGATTCCGGAGAGGGAGCCTGAGAAACGGCTACCACTTCTACGGAAGGCAGCAGGCGCGAAAATTATCCAATCCTGATTTAGGGAGGTAGTGAAAGAAATAATGAACTAGGATTTATCCTGGGATCACAATGGGCTTGATTTGCAAACTCAATTTTAGCGAGGAACGATTGGAGGGCAAGTCTGGTGCCAGCAGCCGCGGTAATTCCAGCTCCAATAGTGTATATTAATGTTCCTGCAGTTATTCGATGCTCGTAGTTGGATTTCTGGAGGTCGCGACAGGGGGTGGCCACGGTCGCCCCAGTCGCCTCCTTCATCCACCTGTTAACGTTGCCCGGGATTTGTTTCTCGGCTTCGGGCTCAGGTATTTTACCCTTTCTATGTATATTCATTTATTCTCTTTTGAGCAAATTATAGTGTTTCAGGCAGGCGTGCGCCGGAATACCTTAGCATGGAATAATCGAACAGGACCGTGTATCCATTATTTGTTGGTTTGAGGGACACGGAAATGGTTAATAGGGATAGTAATTTTCAGGGGAGGCATTAGTATTTAATTTCCAGAGGTGAAATTCTTTGAAATATTAAAGACTAACTTATGCGAAAGCATTTATTTGCCAATAATGTTTTCATTAATCATTGAACGAAAGTTAGGGGATCAAAGACGATCAGATACCGTCCTAGTCTTAACCATAAACGTTGCCGACTAGGGATCGGAGGGCGTGCACATTCCGCCTTCGGCACCTTACGAGAAATCAAAGTCTTTTGGGTTCTGGGGGTAGTATGGTCGCAAGGCTGAAACTTAAAGGAATTGACGGAAGGGCACCACCAGGAGTGGAGCTTGCGGCTCAATTTGACTCAACACGGGAAATCTTACCAGGTCCAGACATAGCGAGGATTGACAGATTGATAGCTCTTTCTTGATTCTATGGGTATTTTTGGTGGTGCATGGCCGTTCTTAGTTGGTGGAGTGATTTGTCTGGTTAATTCCGTTAAACGAACGAGACCTCAGCCTGCTAAATAGTTACCTGTCTTTTCTTTGAGACTTGCTAACTTCTTAGAGGGACGTTGTGTGCAACCACAAGGAAGTTGAGGCAATAACAGGTCTGTGATGCCCTTAGATGTCCTGGGCCGCACGCGTGCTACACTGATACGTACAACGAGGTATATGCATTTTCGCATCGACGCTGCTCCGAAATAGACACAGCTAAATCTTCTAAAATACGTATCGTGCTGCGGATAGATCGTTGAAATTATGAATCTTGAAGGTGGAATTCCTAGTAAGCGCAGGTCATCAACCTGCGTTGATTACGTCCCTGCCCTTTGTACACACCGCCCGTCGCTCCTACCAATTTCGNAGTTCGGTGAACCTCTTGAACTCCCCGGTTTTGGTAGACTTCTTGGTCCGTGGTTACCTCCATGTTCCTGCCGGAACCCG

>2 Euplotes cf mutabilis_ PP648190

TTGGGAACCTCGATCGTCGGCATGCATGTCTAAGTATAAAGGTTACATACAATGAAACTGCGAATGGCTCATTCAAACAGTTATAGTTTATTTGGATTTACACATTAGTTAAATGGATAACCGTAGTAATTCTAGGGCTAATACATGCGTTACGGGGGACTTTACGGAACCCCAGTATTTATTAGATTCAAACCAATATTCCGAAGGTCTACTTGAGATGATTCATGATAACTGATCGAATTGCTGGTCTACCGGCAATAAGTCATTCATGTTTCTGCTTCCCATCAGCTTGATGGTAGTGTATTGGACAACCATGGCATTCACGGGCTATCGGGGGATTAGGGTTCGATTCCGGAGAGGGAGCCTGAGAAACGGCTACCACTTCTACGGAAGGCAGCAGGCGCGAAAATTATCCAATCCTGATTCAGGGAGGTAGTGAAACAAATAATGAACTAGGATTTATCCTGGGGTCACAATGGGCTTGATTTGCAAACTTTATTTAGCGAGGAACAATTGGAGGGCAAGTCTGGTGCCAGCAGCCGCGGTAATTCCAGCTCCAATAGTGTATATTAATGTTCCTGCAGTTATTCGATGCTCGTAGTTGGATTTCTGGAGGCTGAGATCGGAGGGTAGCCAAGGTTACCGCTGAACTCTTCCTTCATCCACCTGTTAACGTTGTCCGGGATTCGTTTCTCGGCTTCGGGCTCAGGCATATACTTTTACCCTTTTTTCTATATTTATTGTTTTGAGTAAATTATAGTGTTTCAGGCAGGCGTGCGCCGGAATACTTTAGCATGGAATAATCGAATTGGACCGTGTATTCTTATTGAATTCTTCCTTATTGTTGGTTCAAGGACACGGAAATGGTTAATAGGGATAGTGTTTTTATTATCAGGGGAGGCATTAGTATTTAATTTCCAGAGGTGAAATTCTTTGAAATATTAAAGACTAACTTATGCGAAAGCATTTATTATTGCCAATAATGTTTTCATTAATCATTGAACGAAAGTTAGGGGATCAAAGACGATCAGATACCGTCCTAGTCTTAACCATAAACGTTGCCGACTAGGGATCGGAGGGCGTGCACATTCCGCCTTCGGCACCTTACGAGAAATCAAAGTCTTTTGGGTTCTGGGGGTAGTATGGTCGCAAGGCTGAAACTTAAAGGAATTGACGGAAGGGCACCACCAGGAGTGGAGCTTGCGGCTCAATTTGACTCAACACGGGAAATCTTACCAGGTCCAGACATAGCGAGGATTGACAGATTGATAGCTCTTTCTTGATTCTATGGGTATTTTATATATATTTTGGTGGTGCATGGCCGTTCTTAGTTGGTGGAGTGATTTGTCTGGTTAATTCCGTTAAACGAACGAGACCTCAGCCTGCTAAATAGTTACCTGTCTTTTCATTTTTTATAATTACGAGACTTGATAACTTCTTAGAGGGACGTTGTGTGCAACCACAAGGAAGTTGAGGCAATAACAGGTCTGTGATGCCCTTAGATGTCCTGGGCCGCACGCGTGCTACACTGATACGTACAACAAGGGGGGTATATGCATTCATGCATCGACGCTGCTCCGAGATAGACACAGCTAAATCTTCTAAAATACGTATCGTGCTGCGGATAGATCGTTGAAATTATGGATCTTGAAGGTGGAATTCCTAGTAAGCGCGGGTCATCAGCCCGCGTTGATTACGTCCCTGCCCTTTGTACACACCGCCCGTCGCTCCTACCAATTTCGAGTGGCTCGGTGAACCTCTTTGGACTGTCGAGCAATCGCGAAATTAGAGTGAACCTGGTCACTTAGAGAAGAGAAGTCCGACCAGTGGATACGT

>3 Euplotes crenosus_ PP648191

TTTAACCTGGTTGTTTCCTGCCAGTAGTCATACGCTTGTCTCAAAGATTAAGCCATGCATGTCTAAGTATAAAGGTTACATACAATGAAACTGCGAATGGCTCATTCAAACAGTTATAGTTTATTTGGATTTACACATTAGTTAAATGGATAACCGTAGTAATTCTAGGGCTAATACATGCGTTACGGGGGACTTTACGGAACCCCAGTATTTATTAGATTCAAACCAATATTCCGAAGGTCTACTTGAGATGATTCATGATAACTGATCGAATTGCTGGTCTACCGGCAATAAGTCATTCATGTTTCTGCTTCCCATCAGCTTGATGGTAGTGTATTGGACAACCATGGCATTCACGGGCTATCGGGGGATTAGGGTTCGATTCCGGAGAGGGAGCCTGAGAAACGGCTACCACTTCTACGGAAGGCAGCAGGCGCGAAAATTATCCAATCCTGATTCAGGGAGGTAGTGAAACAAATAATGAACTAGGATTTATCCTGGGGTCACAATGGGCTTGATTTGCAAACTTTATTTAGCGAGGAACAATTGGAGGGCAAGTCTGGTGCCAGCAGCCGCGGTAATTCCAGCTCCAATAGTGTATATTAATGTTCCTGCAGTTATTCGATGCTCGTAGTTGGATTTCTGGAGGCTGAGATCGGAGGGTAGCCAAGGTTACCGCTGAACTCTTCCTTCATCCACCTGTTAACGTTGTCCGGGATTCGTTTCTCGGCTTCGGGCTCAGGCATATACTTTTACCCTTTTTCTATATTTATTGTTTTGAGTAAATTATAGTGTTTCAGGCAGGCGTGCGCCGGAATACTTTAGCATGGAATAATCGAATTGGACCGTGTATTCTTATTGAATTCTTCCTTATTGTTGGTTCAAGGACACGGAAATGGTTAATAGGGATAGTGTTTTTATTATCAGGGGAGGCATTAGTATTTAATTTCCAGAGGTGAAATTCTTTGAAATATTAAAGACTAACTTATGCGAAAGCATTTATTATTGCCAATAATGTTTTCATTAATCATTGAACGAAAGTTAGGGGATCAAAGACGATCAGATACCGTCCTAGTCTTAACCATAAACGTTGCCGACTAGGGAYCGGAGGGCGTGCACATTCCGCCTTCGGCACCTTACGAGAAATCAAAGTCTTTTGGGTTCTGGGGGTAGTATGGTCGCAAGGCTGAAACTTAAAGGAATTGACGGAAGGGCACCACCAGGAGTGGAGCTTGCGGCTCAATTTGACTCAACACGGGAAATCTTACCAGGTCCAGACATAGCGAGGATTGACAGATTGATAGCTCTTTCTTGATTCTATGGGTATTTTATATATATTTTGGTGGTGCATGGCCGTTCTTAGTTGGTGGAGTGATTTGTCTGGTTAATTCCGTTAAACGAACGAGACCTCAGCCTGCTAAATAGTTACCTGTCTTTTCATTTTTTATAATTACGAGACTTGATAACTTCTTAGAGGGACGTTGTGTGCAACCACAAGGAAGTTGAGGCAATAACAGGTCTGTGATGCCCTTAGATGTCCTGGGCCGCACGCGTGCTACACTGATACGTACAACAAGGGGGGTATATGCATTCATGCATCGACGCTGCTCCGAGATAGACACAGCTAAATCTTCTAAAATACGTATCGTGCTGCGGATAGATCGTTGAAATTATGGATCTTGAAGGTGGAATTCCTAGTAAGCGCGGGTCATCAGCCCGCGTTGATTACGTCCCTGCCCTTTGTACACACCGCCCGTCGCTCCTACCAATTTCGAGTGGCTCGGTGAACCTCTTTGGACTGTCGAGCAATCGCGAAATTAGAGTGAACCTGGTCACTTAGAGGAAGGAGAAGTCGTAACAAGGTTTCCGTAGGTGAACCTGCGGAAGGATCAATCTTAGAAATTTTATACAACCTAAACTCAGCCAGTGCTCCACGTGAGTAAAGCGAACAAAAATAACTTTCTACTTATTAA

>4 Euplotes cf neopolitanus_ PP648192

GCGGTCCGGTCATCGGATCGATCTGGCTGATCCGGCCAGTATATCTGGGTACATACTCTGAAACTGCGAATGGCTCATTCAAACAGTTATAGTTTATTTGATATTCAAACTAATGAAAGTTAGTTTAAATGGATAACCGTAGTAATTCTAGAGCTAATACATGCGTTACGGGGGACTTTACGGAACCCCAGTATTTATTAGATTCAAACCAATATTCCGCAAGGTCTACTGAGATGATTCATGATAACTGATCGAATTGCTGGGTCTACTAGCAATAAATCATTCATGTTTCTGCTTCCCATCAGCTTGATGGTAGTGTATTGGACAACCATGGCGTTCACGGGCTATCGGGGGATTAGGGTTCGATTCCGGAGAGGGAGCCTGATAAACGGCTACCACTTCTACGGAAGGCAGCAGGCGCGAAAATTATCCAATCCTGACTCAGGGAGGTAGTGAAAGAAATAATGAACTAGGATTTATCCTGGGATCACAATGGGCTTGATTTGCAAACTTTACATTTAGCGAGGAACGATTGGAGGGCAAGTCTGGTGCCAGCAGCCGCGGTAATTCCAGCTCCAATAGTGTATATTAATGTTCCTGCAGTTATTCGATGCTCGTAGTTGGATTTCTGAAGTTGAGATGGGAGGGTGGCTATGGCCATCGCTCGTCTCTTCCTTCATCCACCTGTTAGCGAATCCCGGGATTCACTTCTCGGTTTCGGGCTCAGGTACTTTACCATTTTTATATACCTATATTTATATCCTGTAGTCTTTATCGATTATGTGTTATTTCTTCTTTGAGCAAATTATAGTGTTTCAGGCAGGCGTGCGCCGGAATACATTAGCATGGTATAATCGAATTGGACCGTGTATCCATTATTTGGATTCCTCCTTATTTGTTGGTTTAAAGGACACGGAAATGGTTAATAGGGATAGTATTTAATCCAGGGGAGGCATTAGTATTTAATTTCCAGAGGTGAAATTCTTTGAAATATTAAAGACTAACTTATGCGAAAGCATTTATTTTGCCAATAATGTTTTCATTAATCATTGAACGAAAGTTAGGGGATCAAAGACGATCAGATACCGTCCTAGTCTTAACCATAAACGTTGCCGACTAGGGATCGGAGGGCGTGCACATTCCGCCTTCGGCACCTTACGAGAAATCAAAGTCTTTTGGGTTCTGTGGGTAGTATGGTCGCAAGACTGAAACTTAAAGGAATTGACGGAAGGGCACCACCAGGAGTGGAGCTTGCGGCTCAATTTGACTCAACACGGGAAATCTTACCAGGTCCAGACATAGCGAGGATTGACAGATTGATAGCTCTTTCTTGATTCTATGGGTATTTTTGGTGGTGCATGGCCGTTCTTAGTTGGTGGAGTGATTTGTCTGGTTAATTCCGTTAAACGAACGAGACCTCAGCCTGCTAAATAGTTACCTGTCTCTCTTACGAGACTTGATAACTTCTTAGAGGGACTTTGTGTGCAACCACAAGGAAGTTTGAGGCAATAACAGGTCTGTGATGCCCTTAGATGTCCTGGGCCGCACGCGTGCTACACTGATACGTACAACGAGGTATATGTACTTGTACATCAATGCTGCTCCGAAATAGACACAGCTTAATCTTCTAAAATACGTATCGTGCTGCGGATAGATCGTTGAAAATTTTATAATAACAAATTATGAATCTTGAAGGTGGAATTCCTAGTAAGCGCAGGTCATTAGCCTGCGTTGATTACGTCCCTGCCCTTTGTACACACCGCCCGTCGCTCCTACCAATTTCGAGTGGTTTGGTGAACCTCTTTGGACTGTCGAGCAATCGCGAAATTAGAGTGAACCTGATACTAGAGAAGAGAAGTCGACAATTAGTCCGG

>5 Euplotes trisulcatus_ PP648193

CCCGTCGTCATCGATCGTATGGTTGTCCTGCCAGGTACTGGCGTACTATCGAGAAACTGCGAATGGCTGCCTGAACACTGATAGGTTATTTGATATTCAAACTTATATTTTCTTACTAGTGGAAATGGATGACCGTAGTAATTCTAGAGCTAATACATGCGTTACGGGGGACTTTACGGAACCTCAGTATTTATTATTAAACCAGCATTCCGCAAGGTCTACTGAGATGATTCATGATAACTGATCGAATTGCTGGGTCTACCAGCAATAAATCATTCATGTTTCTGCTTCCCATCAGCTTGATGGTCGTGTATTGGACAACCATGGCATTCACGGGCTATCGGGGGATTAGGGTTCGATTCCGGAGAGGGAGCCTGAGAAACGGCTACCACTTCTACGGAAGGCAGCAGGCGCGAAAATTATCCAATCCTGACTCAGGGAGGTAGTGAAAGAAATAATGAACTAGGATTTATCCTGGGATCACAATGGGCTTGATTTGCAAACTCAATTTTAGCGAGGAACAATTGGAGGGCAAGTCTGGTGCCAGCAGCCGCGGTAATTCCAGCTCCAATAGTGTATATAAATGTTCCTGCAGTTATTCGATGCTCGTAGTTGGATTTCTGGAGGTTGCGACAGGAGGGTGGCTATGGCCGCCGCCGGTCGCCTCCTTCATCCACCTGTTAACGTCGTCCGGGATTCATTTCTCGGCTTCGGGCTCAGGTACTTTACCCTTTCTATATAATATTCATTTATTCATCTTCTTTTTGAGCAAATTATAGTGTTTCAGGCAGGCGTGCGCCGGAATACCTTAGCATGGAATAATCGAACTGGACCGTGTATCCATCTTTGTTGGTTTGAGAGACACGGAAATGGTTAATAGGGATAGTACTTTTCAGGGGAGGCATTAGTATTTAATTTCCAGAGGTGAAATTCTTTGAAATATTAAAGACTAACTTATGCGAAAGCATTTATTTTGCCAATAATGTTTTCATTAATCATTGAACGAAAGTTAGGGGATCAAAGACGATCAGATACCGTCCTAGTCTTAACCATAAACGTTGCCGACTAGGGATCGGAGGGCGTGCACATTCCGCCTTCGGCACCTTACGAGAAATCAAAGTCTTTTGGGTTCTGGGGGTAGTATGGTCGCAAGGCTGAAACTTAAAGGAATTGACGGAAGGGCACCACCAGGAGTGGAGCTTGCGGCTCAATTTGACTCAACACGGGAAATCTTACCAGGTCCAGACATAGCGAGGATTGACAGATTGATAGCTCTTTCTTGATTCTATGGGTATTTTTGGTGGTGCATGGCCGTTCTTAGTTGGTGGAGTGATTTGTCTGGTTAATTCCGTTAAACGAACGAGACCTCAGCCTGCTAAATAGTTACCTGTCTTTCTTTTCGAGACTTGTTAACTTCTTAGAGGGACGTTGTGTGCAACCACAAGGAAGTTGAGGCAATAACAGGTCTGTGATGCCCTTAGATGTCCTGGGCCGCACGCGTGCTACACTGATACGTACAACGAGGTATATGCATTTATGCATCGACGCTGCTCCGAAATAGACAAAGCTAAATCTTCTAAAATACGTATCGTGCTGCGGATAGATCGTTGCAATTATGAATCTTGAAGGTGGAATTCCTAGTAAGCGCAGGTCATTAACCTGCGTTGATTACGTCCCTGCCCTTTGTACACACCGCCCGTCGCTACTACCAATTTCGAGTGGCTTGGTGAACCTCTTTGGACTGTCGAGCAATCGCGAAATTAGAGTGAACCTGGTCACTTAGAGAAGTAAAGTCGAACCCCAGCACCGC

>6 Euplotes gracilis_ PP648194

GGGGGCTGTTGCGTCGGCGCGTACAGGTGATCTGCAGTACCTGGTACATCCAACGAGTCTGCGAATGGCTCATTCAGCAGTTATAGTTTATTTGATATTCAAACTTATATTTCATATTAGTTTAAATGGATTACCGTAGTAATTCTAGAGCTAATACATGCGTTACGAGGGACTTTACGGAACCTCAGTATTTATTAGATTTCCAAACCAATATTCCGCAAGGTCTACTGAGATGATTCATGATAACTGATCGAATTGCTGGGTCTACTAGCAATAAATCATTCATGTTTCTGCTTCCCATCAGCTTGATGGTCGTGTATTGGACAACCATGGCATTCACGGGCTATCGGGGGATTAGGGTTCGATTCCGGAGAGGGAGCCTGAGAAACGGCTACCACTTCTACGGAAGGCAGCAGGCGCGAAAATTATCCAATCCTGACTCAGGGAGGTAGTGAAAGAAATAATGAACTAGGATTTATCCTGGGATCACAATGGGCTTGATTTGCAAACTTTATTTAGCGAGGAACAATTGGAGGGCAAGTCTGGTGCCAGCAGCCGCGGTAATTCCAGCTCCAATAGTGTATATTAATGTTCCTGCAGTTATTCGATGCTCGTAGTTGGATTTCTGGAGGTAGAGACAGGAGGGCGGCTATGGCCGCCGCCAGCCTCTTCCTTCATCCACCTGTTAACGTTGCCCGAGATTCATTTCTCGGCTTCGGGCTCAGGTATTTTACCCTTTCTATATATTCATTTATTATCTTATTTTTGAGCAAATTATAGTGTTTCAGGCAGGCGTGCGCCGGAATACATTAGCATGGAATAATCGAACAGGACCGTGATCTTATTTGTTGGTTTCGAAGGACACGGAAATGGTTAATAGGGATAGTAATTTTCAGGGGAGGCATTAGTATTTAATTTCCAGAGGTGAAATTCTTTGAAATATTAAAGACTAACTTATGCGAAAGCATTTATTTTGCCAATAATGTTTTCATTAATCATTGAACGAAAGTTAGGGGATCAAAGACGATCAGATACCGTCCTAGTCTTAACCATAAACGTTGCCGACTAGGGATCGGAGGGCGTGCACATTCCGCCTTCGGCACCTTACGAGAAATCAAAGTATTTGGGTTCTGGGGGTAGTATGGTCGCAAGGCTGAAACTTAAAGGAATTGACGGAAGGGCACCACCAGGGGTGGAGCTTGCGGCTCAATTTGACTCAACACGGGAAATCTTACCAGGTCCAGACATAGCGAGGATTGACAGATTGATAGCTCTTTCTTGATTCTATGGGTATTTTTGGTGGTGCATGGCCGTTCTTAGTTGGTGGAGTGATTTGTCTGGTTAATTCCGTTAAACGAACGAGACCTCAGCCTGCTAACTAGTTACCTGTCTTTCCATTTACGAGACTTGCTAACTTCTTAGAGGGACGTTGTGTGCAACCACAAGGAAGTTGAGGCAATAACAGGTCTGTGATGCCCTTAGATGTCCTGGGCCGCACGCGTGCTACACTGATACGTACAACAAGGTATATGCATTCGTGCATCAATGCTGCTCCGAAATAGACACAGCTAAATCTTCTAAAATACGTATCGTGCTGCGGATAGATCGTTGAAATTATGAATCTTGAAGGCGGAATCCCTAGTAAGCGCAAGTCATCAACTTGCGCTGATTACGTCCCTGCCCTTTGTACACACCGCCCGTCGCTCTACCAATTTCGAGTGGCTCGGTGAACCTCTTTGGACTGTGCMGMATCGGAAATTARGNGAMCTGGWGCTTACAGAAGGAAATCCTGCGTAAAAACAAACACCC

>7 Euplotes muscorum oligomenbrana_n._subsp._ PP648195

CCCGGTGAAATTCAGAACCTGGTTGATCCTGCAGTAGCCTGGTTAGATCCAATGAGTAGCGTGAATGGCTCATTCAAGCAGTTATAGTTTATTTGATATTCAAACTTATTCATATTAGTCTAAATGGATAACCGTAGTAATTCTAGAGCTAATACATGCGTTACGAGGGACTTTACGGAACCTCAGTATTTATTAGATTTCCAAACCAATATTCCGCAAGGTCTACTGAGATGATTCATGATAACTGATCGAATTGCTGGGTCTACTAGCAATAAATCATTCATGTTTCTGCTTCCCATCAGCTTGATGGTCGTGTATTGGACAACCATGGCATTCACGGGCTATCGGGGGATTAGGGTTCGATTCCGGAGAGGGAGCCTGAGAAACGGCTACCACTTCTACGGAAGGCAGCAGGCGCGAAAATTATCCAATCCTGACTCAGGGAGGTAGTGAAAGAAATAATGAACTAGGATTTATCCTGGGATCACAATGGGCTTGATTTGCAAATTTGTATATTTAGCGAGGAACAATTGGAGGGCAAGTCTGGTGCCAGCAGCCGCGGTAATTCCAGCTCCAATAGTGTATATTAATGTTCCTGCAGTTATTCGATGCTCGTAGTTGGATTTCTGGAGGTAGAGACAGGAGGGCGGCTATGGCCGCCGCCAGCCTCTTCCTTCATCCACCTGTTAACGTTGTCCGAGATTCATTTCTCGGCTTCGGGCTCAGGCTTTTACCCTTTCATATATATTCATTTATTAACTTTTTTTTTGAGCAAATTATAGTGTTTCAGGCAGGCGTGCGCCGGAATACATTAGCATGGAATAATCGAACAGGACCGTGATCTTATTTGTTGGTTTCGAAGGACACGGAAATGGTTAATAGGGATAGTAATTTTCAGGGGAGGCATTAGTATTTAATTTCCAGAGGTGAAATTCTTTGAAATATTAAAGACTAACTTATGCGAAAGCATTTATTTTGCCAATAATGTTTTCATTAATCATTGAACGAAAGTTAGGGGATCAAAGACGATCAGATACCGTCCTAGTCTTAACCATAAACGTTGCCGACTAGGGATCGGAGGGCGTGCACATTCCGCCTTCGGCACCTTACGAGAAATCAAAGTATTTTGGGTTCTGGGGGTAGTATGGTCGCAAGGCTGAAACTTAAAGGAATTGACGGAAGGGCACCACCAGGGGTGGAGCTTGCGGCTCAATTTGACTCAACACGGGAAATCTTACCAGGTCCAGACATAGCGAGGATTGACAGATTGATAGCTCTTTCTTGATTCTATGGGTTATTTGGTGGTGCATGGCCGTTCTTAGTTGGTGGAGTGATTTGTCTGGTTAATTCCGTTAAACGAACGAGACCTCAGCCTGCTAACTAGTTACCTGTCTTTCCATTTACGAGACTTGCTAACTTCTTAGAGGGACGTTGTGTGCAACCACAAGGAAGTTGAGGCAATAACAGGTCTGTGATGCCCTTAGATGTCCTGGGCCGCACGCGTGCTACACTGATACGTACAACAAGGTATATGCATTCGTGCATCAATGCTGCTCCGAAATAGACACAGCTAAATCTTTAAAANATACGTATCGTGCTGCGGATAGATCGTTGAAATTATGAATCTTGAAGGCGGAATCCCTAGTAAGCGCAAGTCATCAACTTGCGCTGATTACGTCCCTTCCCTTAGTACACACCGCCCGTCGCTCCTCCCAATTTCGACCCCCTCTAAATATCCTCTTTGGACTTTACATCATTCACCAAAATTAGTAGTCACCTGTAACAGCAGCAAGAAGGGATCCGTCATTGACCTCA

>8 Euplotes paramuscicola_ PP648196

CGGGCTACCGGCGTTCGGACGCGCAGCTGGTGACCAGCAGTTCATGGAGAACGAGCCGCAGCGGGGGGAAGAGAAGAAGTGAGTGCATGTGATATTCAGACTATCTCTCAAAGCTATAGATTGATGGATACCGTAGTACTTCTAGAGCTAATACATGCGTTACGGGGGACTTTACGGAACCTCAGTATTTATTAGATTTCCAAACCAATATTCCGCAGGTCTACTGAGATGATGCATGATAACTGATCGGATTGCTGGGTCTACTAGCAACGCATCATTCATGTTTCTGCTTCCCATCAGCTTGATGGTCGTGTATTGGACAACCATGGCATTCACGGGCTATCGGGGGATTAGGGTTCGATTCCGGAGAGGGAGCCTGAGAAACGGCTACCACTTCTACGGAAGGCAGCAGGCGCGAAAATTATCCAATCCTGACTCAAGGAGGTAGTGAAAGAAATAATGAACTAGGATTTATCCTGGGATCACAATGGGCTTGATTTGCAAACTTTATATTAGCGAGGAACAATTGGAGGGCAAGTCTGGTGCCAGCAGCCGCGGTAATTCCAGCTCCAATAGTGTATATTAATGTTCCTGCAGTTATTCGATGCTCGTAGTTGGATTTCTGGAGGTTGAGACAGGAGGGCGGCTATGGCCGTCGCCAGTCTCTTCCTTCATCCACCTGTTAACGTTGTCCGGGATTAACTTCTCGACTTCGGGCTCAGGTACTTTACCCTTTCTGATATCTTCATTTACATTCATGATATCTTTTGAGCAAATCATAGTGTTTCAGGCAGGCGTGCGCCGGAATACATTAGCATGGAATAATCGAACAGGACCGTGTATCTTATTTGTTGGTTATAAGTACACAGATGGTTAATAGGGATAGTACTTCAGGGGAGGCATTAGTATTTAATTTCCAGAGGTGAAATTCTTTGAAATATTAAAGACTCACTTATGCGAAAGCATTTATTTTGCCAATAATGTTTTCATTAATCAGTGAACGAAAGTTAGGGGATCAAAGACGATCAGATACCGTCCTAGTCTTAACCATAAACGTTGCCGGCTAGGGATCGGAGGGCGTGCACATTCCGCCTTCGGCACCTTACGAGAAATCAAAGTCTTTTGGGTTCTGGGGGTAGTATGGTCGCAAGGCTGAAACTTAAAGGAATTGACGGAAGGGCACCACCAGGGGTGGAGCTTGCGGCTCAATTTGACTCAACACGGGAAATCTTACCAGGTCCAGACATAGCGAGGATTGACAGATTGATAGCTCTTTCTTGATTCTATGGGTATTTTTGGTGGTGCATGGCCGTTCTTAGTTGGTGGAGTGATTTGTCTGGTTAATTCCGTTAAACGAACGAGACCTCAGCCTGCTAACTAGTTGCATGTCTTTCCCATTTCCGAGACTTGCTAACTTCTTAGAGGGACGTTGTGTGCAACCACAAGGAAGTTGAGGCAATAACAGGTCTGTGATGCCCTTAGATGTCCTGGGCCGCACGCGTGCTACACTGATACGTACAACAAGGTATATGCGTTTACGCATCAATGCTGCTCCGAAATAGACACAGCTAAATCTTCTAAAATACGTATCGTGCTGCGGATAGATCGTTGAAATTATGAATCTTGAAGGCGGAATCCCTAGTAAGCGCAGGTCATTAACCTGCGCTGATTACGTCCCTGCCCTTTGTACACCCGCCCGTCGCTCCTACCAATTTCAAGTGGCTCGGGGAACCTCTTTGGACTGTGAACCGGAGCAGGATCAGTAGGGACCCGCAGCAGCTCAGTAGGTATCCGCCGCCCGACGCCG

>9 Euplotes vannus pop 1_ PP648197

GGGTCGGCATCGATTAGGATGACGAAGGATGACGGCCAGTATATGGGTTACAACAATGAAACATGCGAATGGCTCATTCAAACAGTTATAGTTTATTTGGATTTACACATTAGTTAAATGGATAACCGTAGTAATTCTAGGGCTAATACATGCGTTACGGGGGACTTTACGGAACCCCAGTATTTATTAGATTCAAACCAATATTCCGAAGGTCTACTTGAGATGATTCATGATAACTGATCGAATTGCTGGTCTACCGGCAATAAGTCATTCATGTTTCTGCTTCCCATCAGCTTGATGGTAGTGTATTGGACAACCATGGCATTCACGGGCTATCGGGGGATTAGGGTTCGATTCCGGAGAGGGAGCCTGAGAAACGGCTACCACTTCTACGGAAGGCAGCAGGCGCGAAAATTATCCAATCCTGATTCAGGGAGGTAGTGAAACAAATAATGAACTAGGATTTATCCTGGGGTCACAATGGGCTTGATTTGCAAACTTTATTTAGCGAGGAACAATTGGAGGGCAAGTCTGGTGCCAGCAGCCGCGGTAATTCCAGCTCCAATAGTGTATATTAATGTTCCTGCAGTTATTCGATGCTCGTAGTTGGATTTCTGGAGGCTGAGATCGGAGGGTAGCCAAGGTTACCGCTGAACTCTTCCTTCATCCACCTGTTAACGTTGTCCGGGATTCGTTTCTCGGCTTCGGGCTCAGGCATATACTTTTACCCTTTTTTTCTATATTTATTGTTTTGAGTAAATTATAGTGTTTCAGGCAGGCGTGCGCCGGAATACTTTAGCATGGAATAATCGAATTGGACCGTGTATTCTTATTGAATTCTTCCTTATTGTTGGTTCAAGGACACGGAAATGGTTAATAGGGATAGTGTTTTTATTATCAGGGGAGGCATTAGTATTTAATTTCCAGAGGTGAAATTCTTTGAAATATTAAAGACTAACTTATGCGAAAGCATTTATTATTGCCAATAATGTTTTCATTAATCATTGAACGAAAGTTAGGGGATCAAAGACGATCAGATACCGTCCTAGTCTTAACCATAAACGTTGCCGACTAGGGATCGGAGGGCGTGCACATTCCGCCTTCGGCACCTTACGAGAAATCAAAGTCTTTTGGGTTCTGGGGGTAGTATGGTCGCAAGGCTGAAACTTAAAGGAATTGACGGAAGGGCACCACCAGGAGTGGAGCTTGCGGCTCAATTTGACTCAACACGGGAAATCTTACCAGGTCCAGACATAGCGAGGATTGACAGATTGATAGCTCTTTCTTGATTCTATGGGTATTTTATATATATTTTGGTGGTGCATGGCCGTTCTTAGTTGGTGGAGTGATTTGTCTGGTTAATTCCGTTAAACGAACGAGACCTCAGCCTGCTAAATAGTTACCTGTCTTTTCATTTTTTTATAATTACGAGACTTGATAACTTCTTAGAGGGACGTTGTGTGCAACCACAAGGAAGTTGAGGCAATAACAGGTCTGTGATGCCCTTAGATGTCCTGGGCCGCACGCGTGCTACACTGATACGTACAACAAGGGGGGGTATATGCATTCATGCATCGACGCTGCTCCGAGATAGACACAGCTAAATCTTCTAAAATACGTATCGTGCTGCGGATAGATCGTTGAAATTATGGATCTTGAAGGTGGAATTCCTAGTAAGCGCGGGTCATCAGCCCGCGTTGATTACGTCCCTGCCCTTTGTACACACCGCCCGTCGCTCCTACCAATTTCGAGTGGCTCGGTGAACCTCTTTGGACTGTCGAGCAATCGCGAAATTAGAGTGAACCTGGTCACTAGAGAAGAGAGTCCGACCATGACCGGG

>10 Euplotes vannus pop 2_ PP648198

GGCCATAATCGATATAGGTTGACAGACAGGATGATCCTGCCGTGCATATGGATCGTAATCGGACGTATGCGAATGGCTCATTCAAACAGTTATAGTTTATTTGGATTTACACATTAGTTAAATGGATAACCGTAGTAATTCTAGGGCTAATACATGCGTTACGGGGGACTTTACGGAACCCCAGTATTTATTAGATTCAAACCAATATTCCGAAGGTCTACTTGAGATGATTCATGATAACTGATCGAATTGCTGGTCTACCGGCAATAAGTCATTCATGTTTCTGCTTCCCATCAGCTTGATGGTAGTGTATTGGACAACCATGGCATTCACGGGCTATCGGGGGATTAGGGTTCGATTCCGGAGAGGGAGCCTGAGAAACGGCTACCACTTCTACGGAAGGCAGCAGGCGCGAAAATTATCCAATCCTGATTCAGGGAGGTAGTGAAACAAATAATGAACTAGGATTTATCCTGGGGTCACAATGGGCTTGATTTGCAAACTTTATTTAGCGAGGAACAATTGGAGGGCAAGTCTGGTGCCAGCAGCCGCGGTAATTCCAGCTCCAATAGTGTATATTAATGTTCCTGCAGTTATTCGATGCTCGTAGTTGGATTTCTGGAGGCTGAGATCGGAGGGTAGCCAAGGTTACCGCTGAACTCTTCCTTCATCCACCTGTTAACGTTGTCCGGGATTCGTTTCTCGGCTTCGGGCTCAGGCATATACTTTTACCCTTTTTTCTATATTTATTGTTTTGAGTAAATTATAGTGTTTCAGGCAGGCGTGCGCCGGAATACTTTAGCATGGAATAATCGAATTGGACCGTGTATTCTTATTGAATTCTTCCTTATTGTTGGTTCAAGGACACGGAAATGGTTAATAGGGATAGTGTTTTTATTATCAGGGGNAGGCATTAGTATTTAATTTCCAGAGGTGAAATTCTTTGAAATATTAAAGACTAACTTATGCGAAAGCATTTATTATTGCCAATAATGTTTTCATTAATCATTGAACGAAAGTTAGGGGATCAAAGACGATCAGATACCGTCCTAGTCTTAACCATAAACGTTGCCGACTAGGGATCGGAGGGCGTGCACATTCCGCCTTCGGCACCTTACGAGAAATCAAAGTCTTTTGGGTTCTGGGGGTAGTATGGTCGCAAGGCTGAAACTTAAAGGAATTGACGGAAGGGCACCACCAGGAGTGGAGCTTGCGGCTCAATTTGACTCAACACGGGAAATCTTACCAGGTCCAGACATAGCGAGGATTGACAGATTGATAGCTCTTTCTTGATTCTATGGGTATTTTATATATATTTTGGTGGTGCATGGCCGTTCTTAGTTGGTGGAGTGATTTGTCTGGTTAATTCCGTTAAACGAACGAGACCTCAGCCTGCTAAATAGTTACCTGTCTTTTCATTTTTTATAATTACGAGACTTGATAACTTCTTAGAGGGACGTTGTGTGCAACCACAAGGAAGTTGAGGCAATAACAGGTCTGTGATGCCCTTAGATGTCCTGGGCCGCACGCGTGCTACACTGATACGTACAACAAGGGGGGTATATGCATTCATGCATCGACGCTGCTCCGAGATAGACACAGCTAAATCTTCTAAAATACGTATCGTGCTGCGGATAGATCGTTGAAATTATGGATCTTGAAGGTGGAATTCCTAGTAAGCGCGGGTCATCAGCCCGCGTTGATTACGTCCCTGCCCTTTGTACACACCGCCCGTCGCTCCTACCAATTTCGAGTGGCTCGGTGAACCTCTTTGGACTGTCGAGCAATCGCGTAAATTAGAGTGAACACTGTCACGTACGAGAAAGATGATGCCGTCCAATGCCTGG

>11 Euplotes n sp_ PP648199

TTGGCCGGGAGTCTGGCTCTGGACAGGAGTCTACACGGGCCAAGGGAGGCTTTACACATGACACTGCGAATGGCACATTAAACAGTTATAGTGTATGTGATATTCAGACTATACTCGTATAGTTTAAATGGATAACCGTAGTAATTCTAGAGCTAATACATGCGTTACGGGGGACTTCACGGAACCTCAGTATTTATTAGATTCCAAACCAATATTCCGCAAGGTCTACTGAGATGATTCATGATAACTGATCGGACTGCTGGGTCTACTAGCAGTGAATCATTCATGTTTCTGCTTCCCATCAGCTTGATGGTCGTGTATTGGACAACCATGGCATTCACGGGCTATCGGGGGATTAGGGTTCGATTCCGGAGAGGGAGCCTGAGAAACGGCTACCACTTCTACGGAAGGCAGCAGGCGCGAAAATTATCCAATCCTGACTCAGGGAGGTAGTGAAAGAAATAATGAACTAGGATTTATCCTGGGATCACAATGGGCTTGATTTGCAAACTTTATATTAGCGAGGAACAATTGGAGGGCAAGTCTGGTGCCAGCAGCCGCGGTAATTCCAGCTCCAATAGTGTATATTAATGTTCCTGCAGTTATTCGATGCTCGTAGTTGGATTTCTGGAGGTTGAGACAGGAGGGCGGCTATGGCCGCCGCCAGTCTCTTCCTTCATCCACCTGTTAACGTTGTCCGGGATTCACTTCTCGACTTCGGGCTCAGGTACTTTACCCTTTTCTTATATCTTCTTTGATATCTTTTGAGCAAATCATAGTGTTTCAGGCAGGCGTGCGCCGGAATACATTAGCATGGAATAATCGAACAGGACCGTGTGTCTCCTTTGTTGGTTATGAACACACAGATGGTTAATAGGGATAGTACTTCAGGGGAGGCATTAGTATTTAATTTCCAGAGGTGAAATTCTTTGAAATATTAAAGACTAACTTATGCGAAAGCAGGTATTTTGCCAATAATGTTTTCATTAATCATTGAACGAAAGTTAGGGGATCAAAGACGATCAGATACCGTCCTAGTCTTAACCATAAACGTTGCCGGCTAGGGATCGGAGGGCGTGCACATTCCGCCTTCGGCACCTTACGAGAAATCAAAGTCTTTTGGGTTCTGGGGGTAGTATGGTCGCAAGGCTGAAACTTAAAGGAATTGACGGAAGGGCACCACCAGGGGTGGAGCTTGCGGCTCAATTTGACTCAACACGGGAAATCTTACCAGGTCCAGACATAGCGAGGATTGACAGATTGATAGCTCTTTCTTGATTCTATGGGTATTTTTGGTGGTGCATGGCCGTTCTTAGTTGGTGGAGTGATTTGTCTGGTTAATTCCGTTAAACGAACGAGACCTCAGCCTGCTAACTAGTTGCATGTCTTTCCCATTTCCGAGACTTGCAATCTTCTTAGAGGGACGTTGTGTGCAACCACAAGGAAGTTGAGGCAATAACAGGTCTGTGATGCCCTTAGATGTCCTGGGCCGCACGCGTGCTACACTGATACGTACAACAAGGTATATGCATCTCGCATCAATGCTGCTCCGAAATAGACACAGCTAAATCTTCTAAAATACGTATCGTGCTGCGGATAGATCGTTGAAATTATGAATCTTGAAGGCGGAATCCCTAGTAAGCGCAGGTCATTAACCTGCGCTGATTACGTCCCTGCCCTTTGTACACACCGCCCGTCGCTCCTACCAATTTCGAGTGGCTCGGGGAACCTCTTTGGACTRCGACCATCGAGCAATTTAAAACTCCCTGGGCCCCGTTAAAATCCCTAGTACTAAGCCCGGAAACCCG

>12 Euplotes sp_ PP648200

TCCGAGAGGAATCTGCTTGTACCAGGAAGCTACCACGGCCCAAGAAGCTAAAACAAATCTGCGAATGGCTCATTAAACAATAATAGTGTATGTGATATTCAGACTATACTCGTATAGTTTAAATGGATAACCGTAGTAATTCTAGAGCTAATACATGCGTTACGGGGGACTTCACGGAACCTCAGTATTTATTAGATTCCAAACCAATATTCCGCAAGGTCTACTGAGATGATTCATGATAACTGATCGGACTGCTGGGTCTACTAGCAGTGAATCATTCATGTTTCTGCTTCCCATCAGCTTGATGGTCGTGTATTGGACAACCATGGCATTCACGGGCTATCGGGGGATTAGGGTTCGATTCCGGAGAGGGAGCCTGAGAAACGGCTACCACTTCTACGGAAGGCAGCAGGCGCGAAAATTATCCAATCCTGACTCAGGGAGGTAGTGAAAGAAATAATGAACTAGGATTTATCCTGGGATCACAATGGGCTTGATTTGCAAACTTTATATTAGCGAGGAACAATTGGAGGGCAAGTCTGGTGCCAGCAGCCGCGGTAATTCCAGCTCCAATAGTGTATATTAATGTTCCTGCAGTTATTCGATGCTCGTAGKTGGATTTCTGGAGGTTGAGACAGGAGGGCGGCTATGGCCGCCGCCAGTCTCTTCCTTCATCCACCTGTTAACGTTGTCCGGGATTCACTTCTCGACTTCGGGCTCAGGTACTTTACCCTTTTCTTATATCTTCTTTGATATCTTTTGAGCAAATCATAGTGTTTCAGGCAGGCGTGCGCCGGAATACATTAGCATGGAATAATCGAACAGGACCGTGTGTCTCCTTTGTTGGTTATGAACACACAGATGGTTAATAGGGATAGTACTTCAGGGGAGGCATTAGTATTTAATTTCCAGAGGTGAAATTCTTTGAAATATTAAAGACTAACTTATGCGAAAGCAGGTATTTTGCCAATAATGTTTTCATTAATCATTGAACGAAAGTTAGGGGATCAAAGACGATCAGATACCGTCCTAGTCTTAACCATAAACGTTGCCGGCTAGGGATCGGAGGGCGTGCACATTCCGCCTTCGGCACCTTACGAGAAATCAAAGTCTTTTGGGTTCTGGGGGTAGTATGGTCGCAAGGCTGAAACTTAAAGGAATTGACGGAAGGGCACCACCAGGGGTGGAGCTTGCGGCTCAATTTGACTCAACACGGGAAATCTTACCAGGTCCAGACATAGCGAGGATTGACAGATTGATAGCTCTTTCTTGATTCTATGGGTATTTTTGGTGGTGCATGGCCGTTCTTAGTTGGTGGAGTGATTTGTCTGGTTAATTCCGTTAAACGAACGAGACCTCAGCCTGCTAACTAGTTGCATGTCTTTCCCATTTCCGAGACTTGCAATCTTCTTAGAGGGACGTTGTGTGCAACCACAAGGAAGTTGAGGCAATAACAGGTCTGTGATGCCCTTAGATGTCCTGGGCCGCACGCGTGCTACACTGATACGTACAACAAGGTATATGCATCTCGCATCAATGCTGCTCCGAAATAGACACAGCTAAATCTTCTAAAATACGTATCGTGCTGCGGATAGATCGTTGAAATTATGAATCTTGAAGGCGGAATCCCTAGTAAGCGCAGGTCATTAACCTGCGCTGATTACGTCCCTGCCCTTTGTACACACCGCCCGTCGCTCCTACCAATTTCGAGTGGCTCGGTGAACCTCTTTGGACTGTCGACMATCGTGAATTAAAGGGACGGCCGTGGTTAGCTTCGTTGGTGCCCGCCCCGGAAAGCT

>13 Euplotidium itoi_ PP648201

CCGGAGGGGGTCTGCTGCAGGATCACTATTGATCCTTCTGCAGGTTCACCTACTGATCCTTCTGCAGGTTCACCTACTGATCCTTCTGCAGGTTCACCTACTGATCCTTCTGCAGGATCACCTACTGATCTTGCTGCAGGATCAGCTATACTACTGCCTGCTGGATCGGCCATTATTCATCCTATAACAAACTCATTTTCCTAAGCTGCTATAGTGTCGATTCGTAATACCTCATCAAATCCCTGGGCAATGCCTGCGATAAATCATTCAAGTATCTGCCCCATCAGCTTGTTGGTAGTGTATTGGACTACCATGGCGTTCACGGGTAACGGAGGATTAGGGTTCGATTCCGGAGAGGGAGCCTGAGAAACGGCTACCACTTCTACGGAAGGCAGCAGGCGCGTAAATTACCCAATCCTGATTCAGGGAGGTAGTGACAAGAAATAACAGACCGGAGCCTCGTGCACCGGGGTTGCAATGAGAACAATTTAAACTCCTTAATGAGGACCAATTGGAGGGCAAGTCTGGTGCCAGCAGCCGCGGTAATTCCAGCTCCAATAGCGTATATAAAAGTTGTTGCAGTTAAAAAGCTCGTAGTTGGATTTCTGGATATGCGCTGATGTCGGCCGTGTGCTCGTGCAGATGCGCGTTTCCATCCTTCTGTTAACGTTTCTTGGTATTCATTTACTGGTTTCGGGCTCAGATATTTTACCTTGAGAAAATTAGAGTGTTTCAGGCAGGCTTGCGCCGGAATACATTAGCATGGAATAATAGAATAGGACTACGGTTCCTTTTGTTGGTTTGAGGGCCGAAGTAATGGTTAATAGGGATAGTTGGGGGCATTAGTATTTAATTGTCAGAGGTGAAATTCTTTGATTTGTTAAAGACTAACCTATGCGAAAGCATTTGCCAAGGATGTTTTCATTAATCAAGAACGAAAGTTAGGGGATCAAAGACGATCAGATACCGTCCTAGTCTTAACCATAAACTATGCCGACTAGGGATTGGAGGCGTGCGAAATCCGCCTTCAGCACCTTATGAGAAATCAAAGTCTTTGGGTTCTGGGGGGAGTATGGTCGCAAGGCTGAAACTTAAAGGAATTGACGGAAGGGCACCACCAGGAGTGGAGCTTGCGGCTTAATTTGACTCAACACGGGAAAACTTACCAGGTCCAGACATAGTAAGGATTGACAGATTGATAGCTCTTTCTTGATTCTATGGGTGGTGGTGCATGGCCGTTCTTAGTTGGTGGAGTGATTTGTCTGGTTAATTCCGTTAACGAACGAGACCTTAGCCTACTAAATAGTTACTACTCCTTGGGAGTATTTAACTTCTTAGAGGGACTTTGTGTGCAACCACAAGGAAGTTTGAGGCAATAACAGGTCTGTGATGCCCTTAGATGTCCTGGGCCGCACGCGTGCTACACTGACGCGTACACCGAGTATTCCTGCTCCGCGAGGCAGCAGGTAATCTACAATACGCGTCGTGATGGGGATAGATCTTTGGAATTATAGATCTTGAACGAGGAATTCCTAGTAAGCGTAAATCATTAGTTTGCGCTGATTAAGTCCCTGCCCTTTGTACACACCGCCCGTCGCTCCTACCGATTTCGAGTGATCCGGTGAACCTTTCGAACTGTGAGCGCAAGCCAGCGGGATGTAGTAGTAGACCCTGCAGCTAGACAGTAGGGATTCTTAGACCCCAATTCA
