# Supplementary figures and images for: Evolutionary patterns of the SSU rRNA (V4 region) secondary structure in genus Euplotes (Ciliophora, Spirotrichea): insights into cryptic species and primitive traits
Source: PeerJ. 2025 Jan 23;13:e18852. doi: 10.7717/peerj.18852 (PMC11766670; doi:10.7717/peerj.18852)

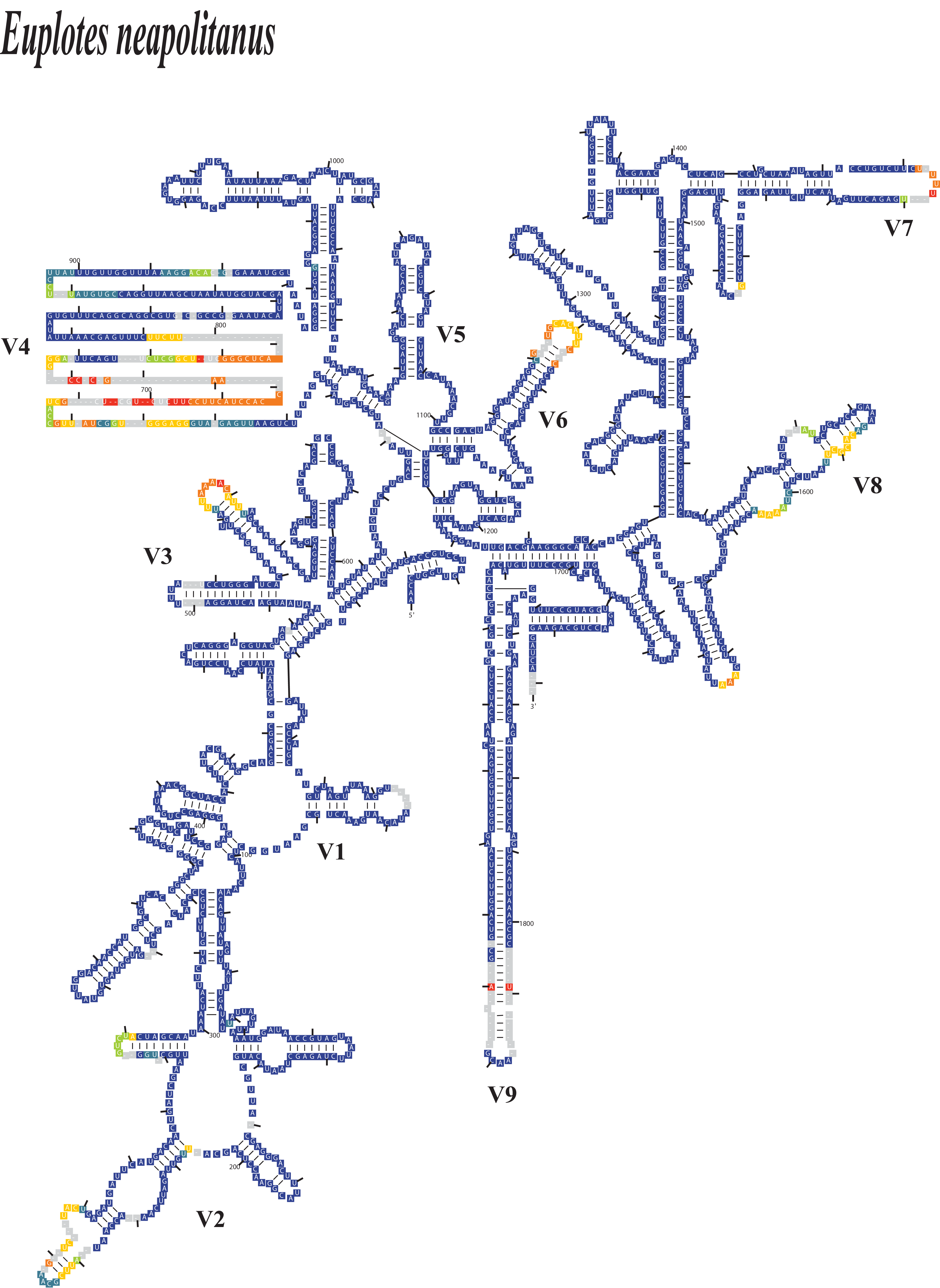

Supplement: Supplemental Information 4 — The color coding represents alignment confidence values (low to high: dark orange, orange, yellow, green, cyan, blue), with gaps indicated in gray. Lower confidence values correspond to nucleotide base changes at specific positions, while gaps indicate potential deletions. [file peerj-13-18852-s004.png]

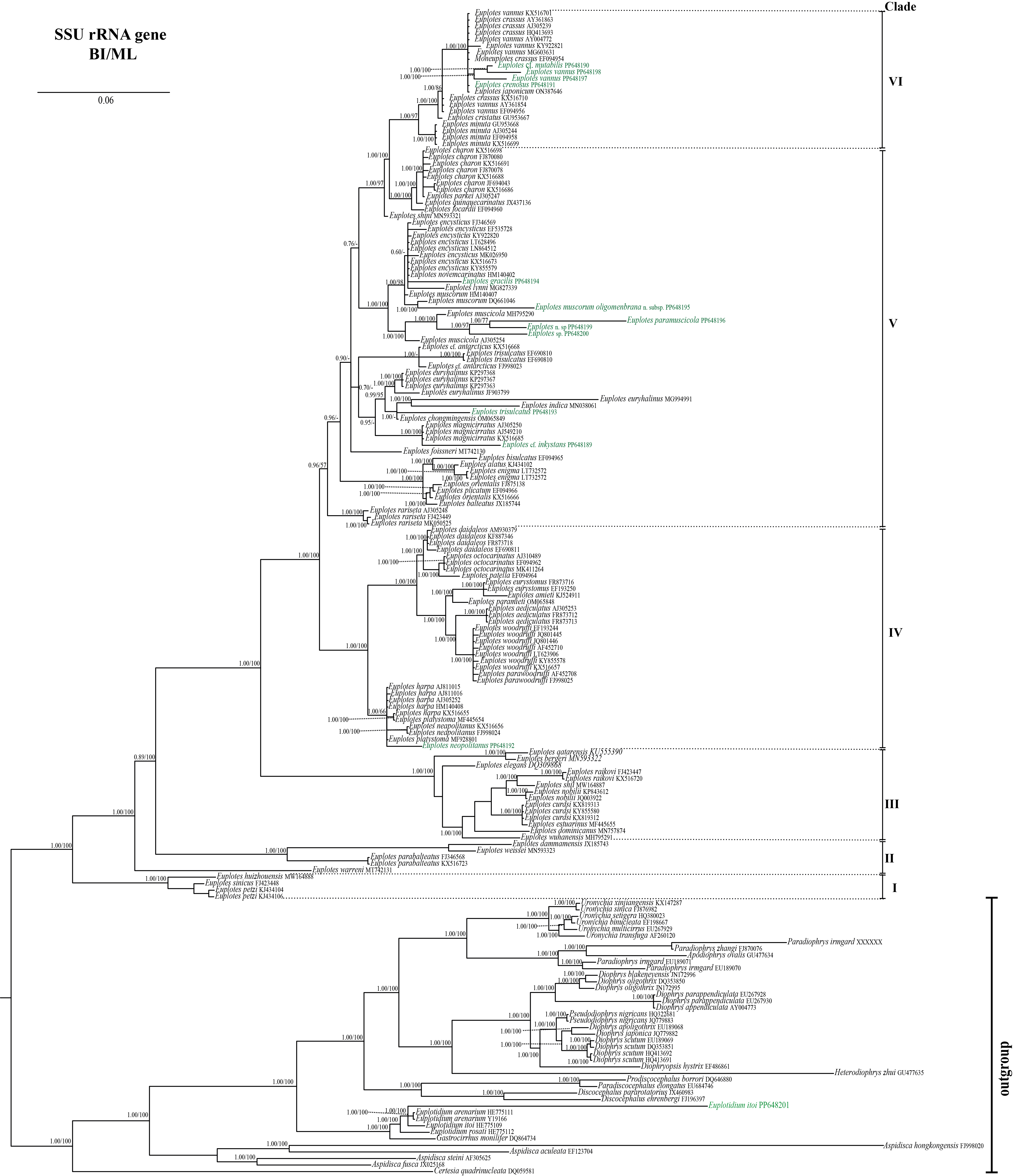

Supplement: Supplemental Information 5 — Node values represent statistical support from BI/ML tree, while node value under 50% and unsynchronized branches are represented with “- “. Newly sequenced species are marked in green. [file peerj-13-18852-s005.png]

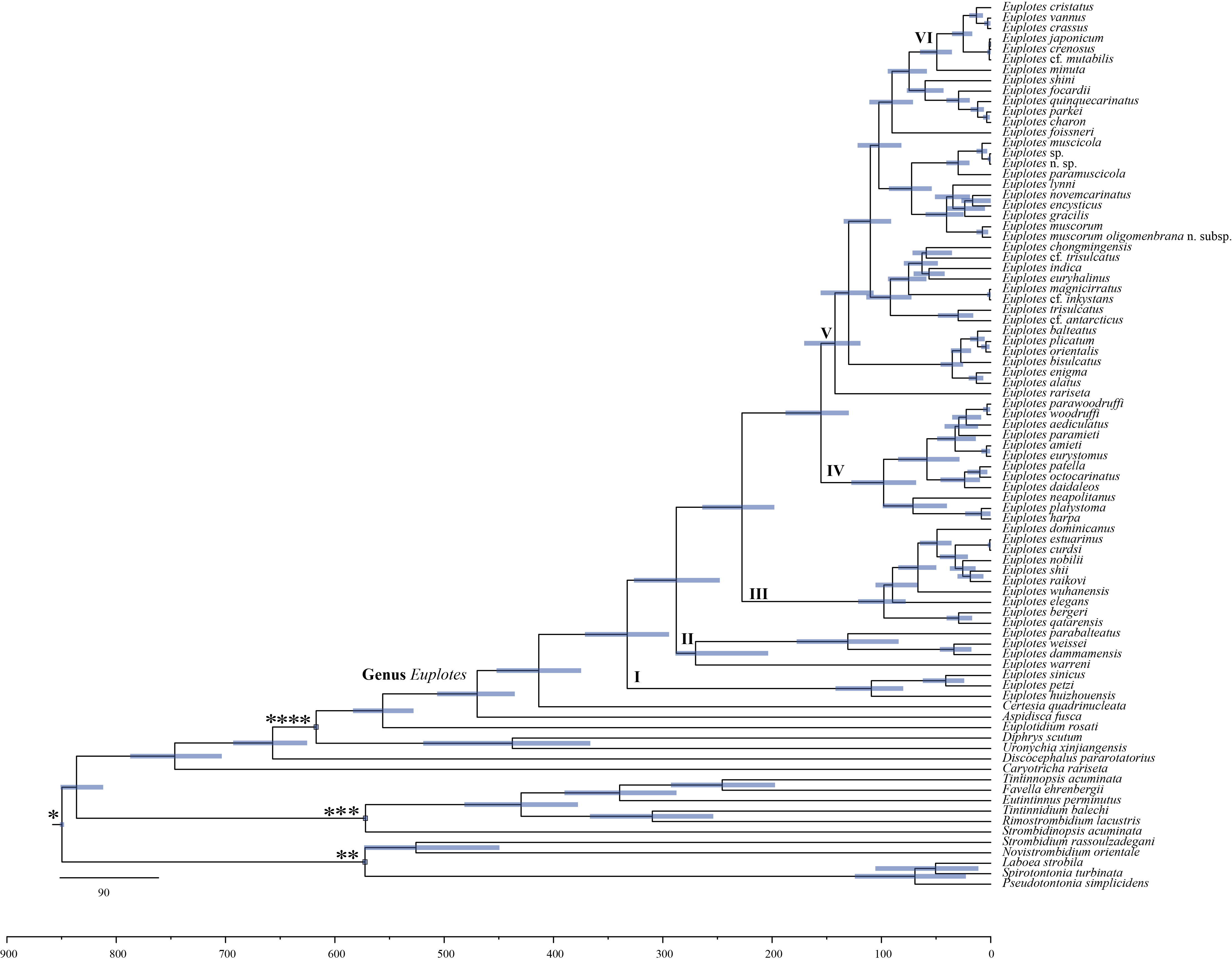

Supplement: Supplemental Information 6 — The 95% credibility intervals are indicated by bars in each node, and horizontal lines show the dating in million years. The parameter shown in Article S1. [file peerj-13-18852-s006.png]

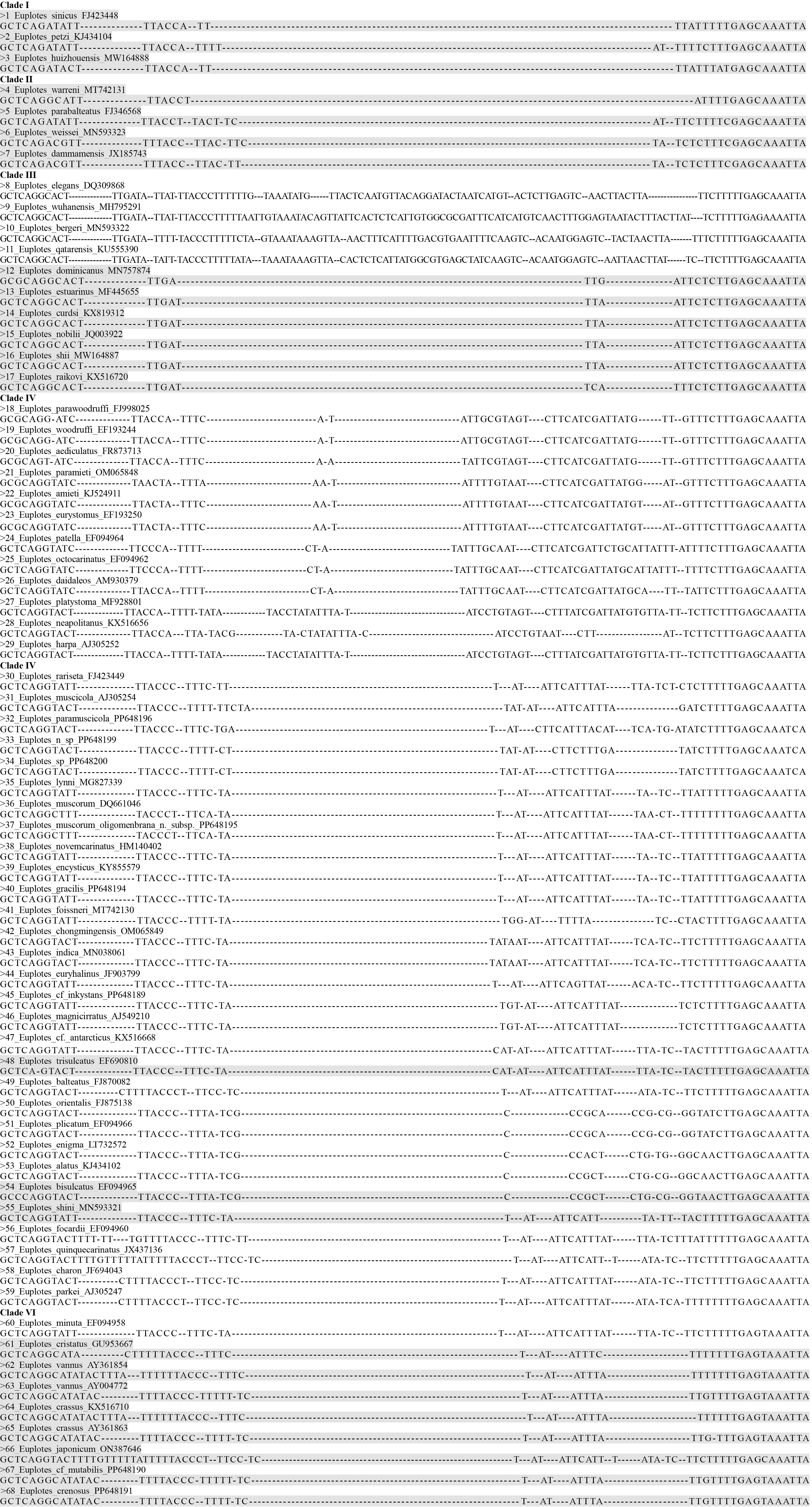

Supplement: Supplemental Information 7 [file peerj-13-18852-s007.png]

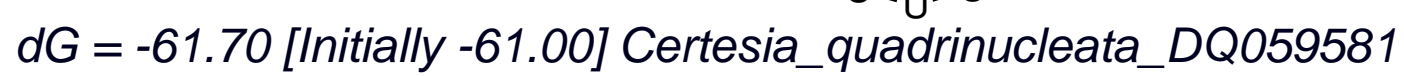

Supplement: Supplemental Information 9 [file peerj-13-18852-s009.pdf]

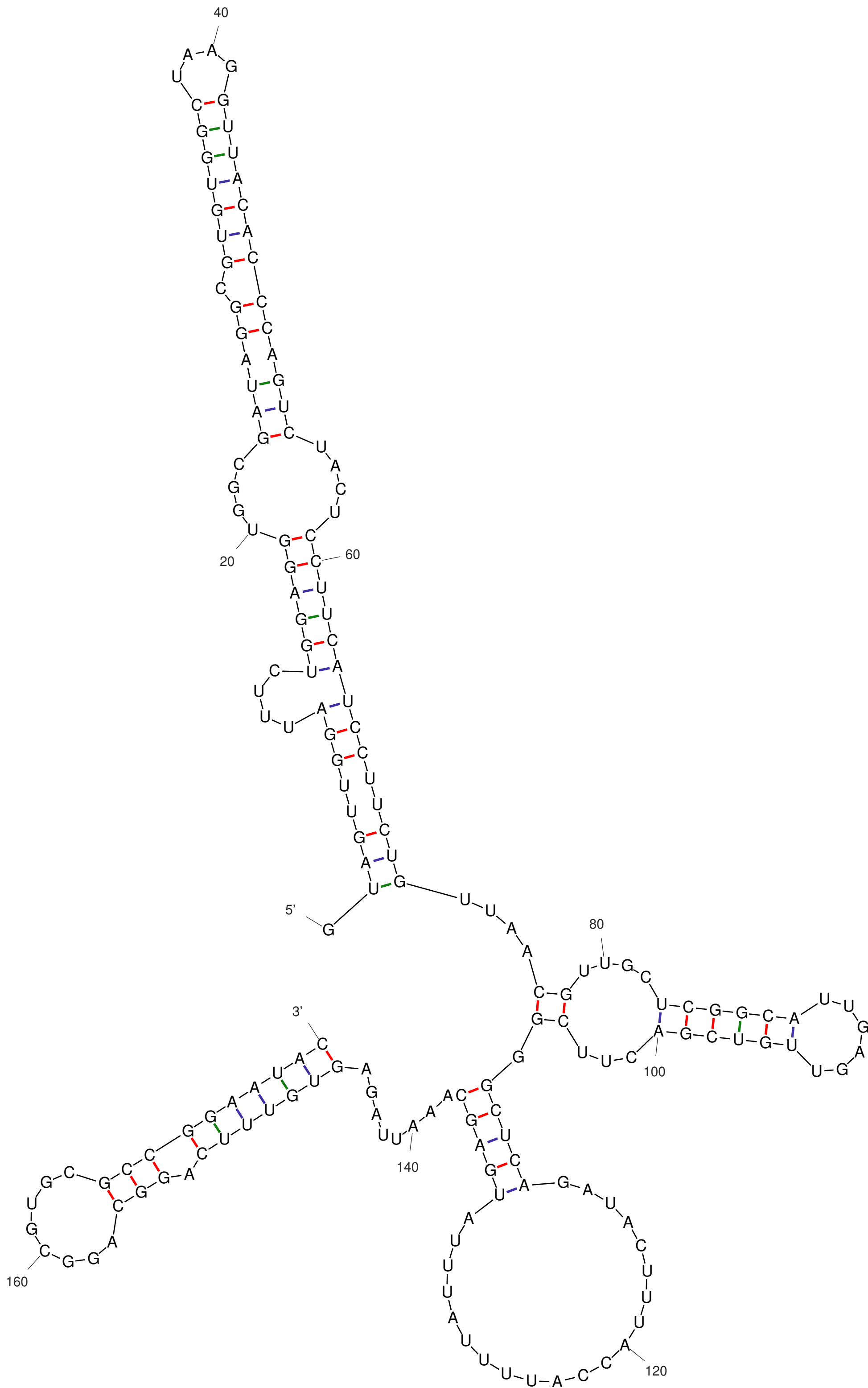

dG = -48.20 [Initially -48.10] *Euplotes huizhouensis*\_MW164888

Supplement: Supplemental Information 10 [file peerj-13-18852-s010.pdf]

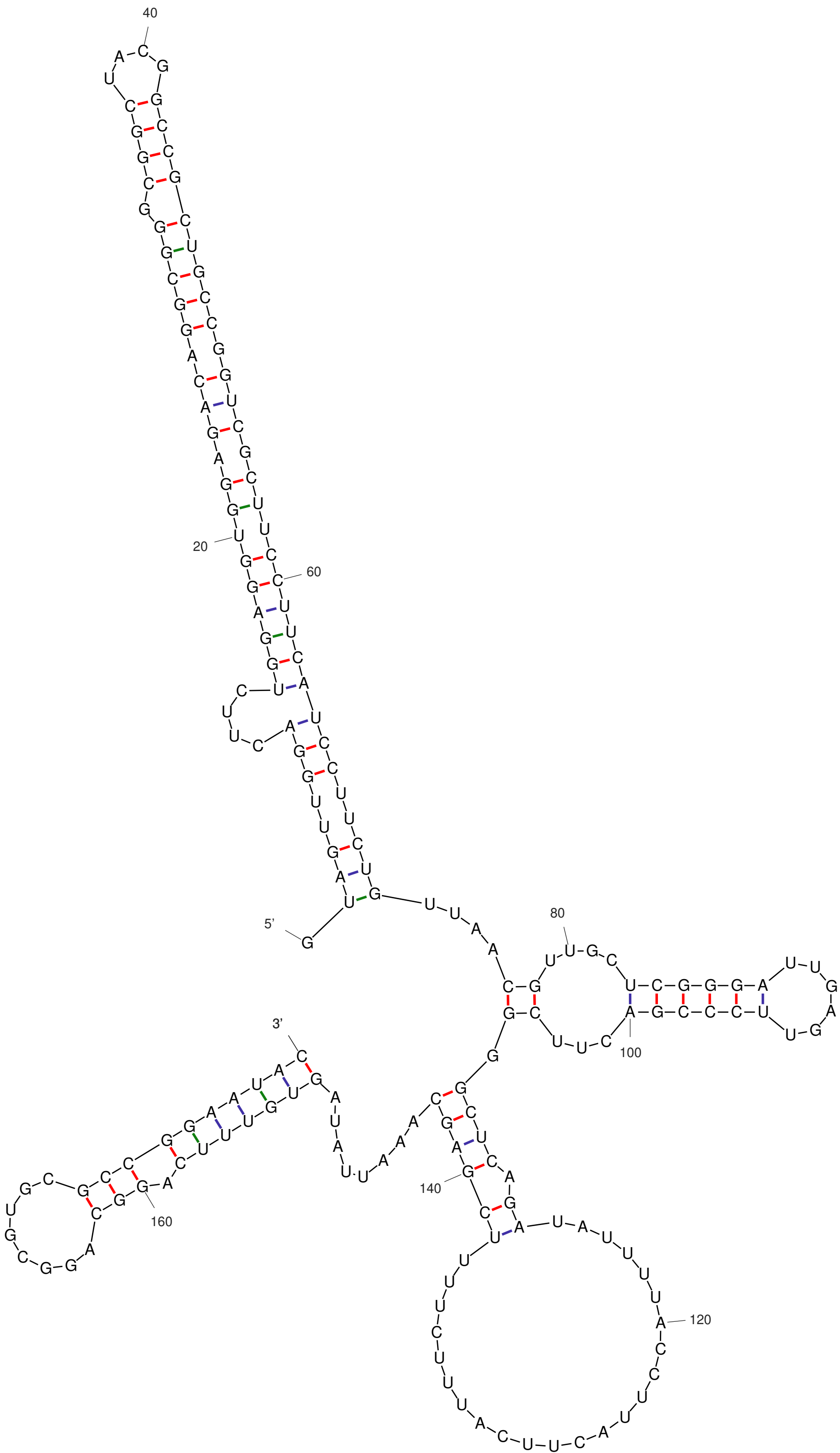

$dG = -62.20$  [Initially -62.10] *Euplotes parabalteatus*\_FJ346568

Supplement: Supplemental Information 11 [file peerj-13-18852-s011.pdf]

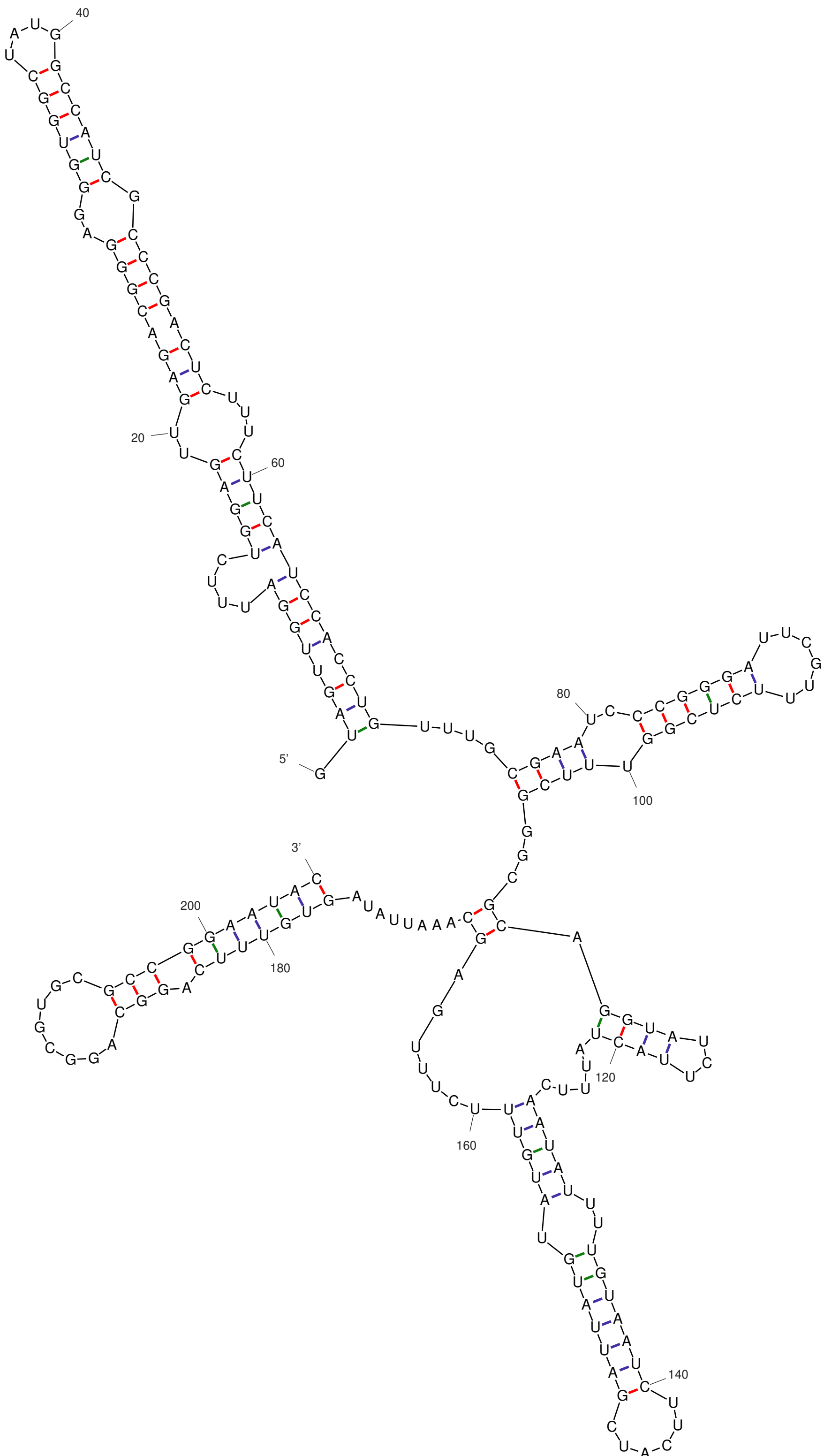

$dG = -56.95$  [Initially -59.90] *Euplotes amieti*\_KJ524911

Supplement: Supplemental Information 12 [file peerj-13-18852-s012.pdf]
