# Supplementary material for: Evolutionary patterns of the SSU rRNA (V4 region) secondary structure in genus Euplotes (Ciliophora, Spirotrichea): insights into cryptic species and primitive traits
Source: PeerJ. 2025 Jan 23;13:e18852. doi: 10.7717/peerj.18852 (PMC11766670; doi:10.7717/peerj.18852)
Supplement: Supplemental Information 8 [file peerj-13-18852-s008.docx]

**Supplemental materials and methods**

1. *Dataset assembly and alignments*

The study utilized datasets that included both newly generated sequences (*Table 1*) and published species from the genus Euplotes that obtained from the NCBI GenBank database (*Table S2*). Several species from related genera were selected as outgroups, including Paradiophrys, Apodiophrys, Diophrys, Pseudodiophrys, Diophryopsis, Heterodiophrys, Uronychia, Euplotidium, Gastrocirrhus, Certesia, Aspidisca, Prodiscocephalus, and Discocephalus. These datasets were aligned using MAFFT ver. 7.0 with G-INS-I as Iterative refinement methods (Katoh and Standley, 2013). After alignment, the sequences were refined and masked using G-blocks ver. 0.91b with allowed gap within the final result (Castresana, 2000), resulting in the alignment of 178 sequences with 1,757 molecular characters for further analysis.

1. *Phylogenetic analysis*

Bayesian inference (BI) analyses were performed with MrBayes v.2.1.7 ([Ronquist and Huelsenbeck, 2003](https://www.sciencedirect.com/science/article/pii/S1055790312001467#b0195)) using the GTR + I + G model selected by JModeltest v.2.1.7 (Darriba et al. 2012) according to the AIC criterion. Markov chain Monte Carlo (MCMC) simulations were run with two sets of four chains using the default settings: chain length 1,000,000–3,000,000 generations, with trees sampled every 100 generations. The first 25% of sampled trees were discarded as burn-in. All remaining trees were used to calculate posterior probabilities (PPs) using a majority rule consensus. Maximum likelihood (ML) trees were constructed with PhyML 3.0 (Guindon et al., 2010). The reliability of internal branches was assessed using nonparametric bootstrapping with 1000 replicates. Phylogenetic trees were visualized with Figtree 1.4.3 (Rambaut, 2014)

1. *Molecular dating and diversification analysis*

In this study, representative taxa from the genus *Euplotes* (*Table S1*) and several members of related group were aligned using MAFFT version 7.0 (Katoh and Standley, 2013) (*Figure S3*). To enhance the quality of the alignment, G-blocks version 0.91b (Castresana, 2000) were used. The most suitable evolutionary model for the dataset was determined using jModelTest version 2.17 (Darriba et al., 2015). The preparation of the XML file was conducted in BEAUti ver. 2.4.7 (Drummond et al., 2012) with the following settings: (i) Calibrated Yule model, (ii) GTR+Γ(=0.4740), (iii) Four gamma categories for substitution rate heterogeneity, (iv) Random Local Clock, (v) Clock rate prior assuming gamma with alpha value : 0.05 and beta value 10, and (vi) Yule birth rate, with the shape parameter set to 0.001 and the scale parameter to 1000.

For calibration purposes, as there is no fossil record for *Euplotes*, a calibration node based on Fernanes and Schrago (2019), was utilized. It predicted that Spirotrichea diverged around 850 million years ago (Mya), Euplotia diverged around 617 Mya, Hypotrichia and Choreotrichia diverged around 572 Mya. The Markov Chain Monte Carlo (MCMC) analyses were initiated from a random seed, running for 600,0000 generations, with trees and all other parameters saved every 10,000th iteration. BEAST ver. 2.4.7 (Drummond et al., 2012) was employed to predict the divergence time. The quality of the MCMC analysis was assessed using Tracer version 1.6 (Drummond and Rambaut, 2007). Finally, the final maximum credibility tree was generated in TreeAnnotator ver. 1.8.1 (Drummond and Rambaut, 2007), following the exclusion of the first 10% of sampled trees.

**References:**

Castresana J. 2000. Selection of conserved blocks from multiple alignments for their use in phylogenetic analysis.*Molecular Biology and Evolution* 17(4):540–552

DOI 10.1093/oxfordjournals.molbev.a026334.

Darriba D, Taboada GL, Doallo R, Posada D. 2012. JModelTest 2: more models, new heuristics and high-performance computing. *Nature Methods* 9(8):772

DOI 10.1038/nmeth.2109.

Drummond AJ, Rambaut A. 2007. BEAST: bayesian evolutionary analysis by sampling trees. *BMC*

*Evolutionary Biology* 7(1):1–8 DOI 10.1186/1471-2148-7-214.

Drummond AJ, Suchard MA, Xie D, Rambaut A. 2012. Bayesian phylogenetics with BEAUti and

the BEAST 1.7. *Molecular Biology Evolution* 29 (8):1969–1973 DOI 10.1093/molbev/mss075.

Fernandes NM, Schrago CG. 2019. A multigene timescale and diversiﬁcation dynamics of

Ciliophora evolution*. Molecular Phylogenetics and Evolution* 139:106521

DOI 10.1016/j.ympev.2019.106521.

Guindon S, Dufayard J-F, Lefort V, Anisimova M, Hordijk W, Gascuel O. 2010. New algorithms and methods to estimate maximum-likelihood phylogenies: assessing the performance of

PhyML 3.0. *Systematic Biology* 59(3):307–321 DOI 10.1093/sysbio/syq010.

Katoh K, Standley DM. 2013. MAFFT multiple sequence alignment software version 7: improvements in performance and usability. *Molecular Biology and Evolution* 30(4):772–780

DOI 10.1093/molbev/mst010.

Ronquist F, Huelsenbeck JP. 2003. MrBayes 3: bayesian phylogenetic inference under mixed

models. *Bioinformatics* 19(12):1572–1574DOI 10.1093/bioinformatics/btg180.
